# Antimicrobial Action and Reversal of Resistance in MRSA by Difluorobenzamide Derivatives Targeted at FtsZ

**DOI:** 10.3390/antibiotics9120873

**Published:** 2020-12-05

**Authors:** Wern Chern Chai, Jonathan J. Whittall, Di Song, Steven W. Polyak, Abiodun D. Ogunniyi, Yinhu Wang, Fangchao Bi, Shutao Ma, Susan J. Semple, Henrietta Venter

**Affiliations:** 1 Health and Biomedical Innovation, Clinical and Health Sciences, University of South Australia, SA 5000 Adelaide, Australia; wern_chern.chai@mymail.unisa.edu.au (W.C.C.); Jon.Whittall@unisa.edu.au (J.J.W.); Steven.Polyak@unisa.edu.au (S.W.P.); Susan.Semple@unisa.edu.au (S.J.S.); 2 Department of Medicinal Chemistry, Key Laboratory of Chemical Biology (Ministry of Education), School of Pharmaceutical Sciences, Cheeloo College of Medicine, Shandong University, Jinan 250012, China; sondy321@163.com (D.S.); wangyinhu@lcu.edu.cn (Y.W.); yahe1111@163.com (F.B.); mashutao@sdu.edu.cn (S.M.); 3 Australia Centre for Antimicrobial Resistance Ecology, School of Animal and Veterinary Sciences, University of Adelaide, Roseworthy Campus, SA 5371 Roseworthy, Australia; david.ogunniyi@adelaide.edu.au; 4 School of Pharmacy, Liaocheng University, Liaocheng 252000, China; 5 Quality Use of Medicines and Pharmacy Research Centre, Clinical and Health Sciences, University of South Australia, SA 5000 Adelaide, Australia

**Keywords:** antimicrobial resistance, antimicrobial development, reversing resistance, FtsZ inhibitors, 3-methoxybenzamide, methicillin resistant *Staphylococcus aureus*

## Abstract

The bacterial cell division protein, FtsZ, has been identified as a target for antimicrobial development. Derivatives of 3-methoxybenzamide have shown promising activities as FtsZ inhibitors in Gram-positive bacteria. We sought to characterise the activity of five difluorobenzamide derivatives with non-heterocyclic substituents attached through the 3-oxygen. These compounds exhibited antimicrobial activity against methicillin resistant *Staphylococcus aureus* (MRSA), with an isopentyloxy-substituted compound showing modest activity against vancomycin resistant *Enterococcus faecium* (VRE). The compounds were able to reverse resistance to oxacillin in highly resistant clinical MRSA strains at concentrations far below their MICs. Three of the compounds inhibited an *Escherichia coli* strain lacking the AcrAB components of a drug efflux pump, which suggests the lack of Gram-negative activity can partly be attributed to efflux. The compounds inhibited cell division by targeting *S. aureus* FtsZ, producing a dose-dependent increase in GTPase rate which increased the rate of FtsZ polymerization and stabilized the FtsZ polymers. These compounds did not affect the polymerization of mammalian tubulin and did not display haemolytic activity or cytotoxicity. These derivatives are therefore promising compounds for further development as antimicrobial agents or as resistance breakers to re-sensitive MRSA to beta-lactam antibiotics.

## 1. Introduction

Antimicrobial resistance is a major healthcare issue with multidrug resistant organisms such as methicillin resistant *Staphylococcus aureus* (MRSA) being a particular concern [1,2]. To ensure this is strategically and urgently addressed, the World Health Organization (WHO) published a Global Action Plan in 2015 [3]. One of the five objectives of this multifaceted plan is to increase the investment in development of new antimicrobials with novel mechanisms of action.

One potential drug target for new antimicrobials is the bacterial cell division protein known as filamentous temperature sensitive Z-ring (FtsZ) protein. This protein represents an attractive target for development of novel antimicrobial agents, as it plays an essential role in bacterial cell division in both Gram-positive and Gram-negative bacteria [4] and is unique to bacteria. The first step of bacterial cell division (septation) is coordinated by a complex of proteins collectively named the divisome. Monomers of FtsZ polymerize into filaments at the division site in the presence of GTP that is hydrolysed to GDP. These protofilaments come together through a process described as treadmilling [5] to form a dynamic ring structure called the Z-ring at the mid-cell. This facilitates the recruitment of additional division proteins, including enzymes involved in cell wall synthesis and remodelling. The divisome complex then constricts the cell membrane and facilitates progressive insertion of the new septal cell wall, ultimately dividing the cell [5].

The FtsZ protein is a polymerizing GTPase with a structure that consists of two globular subdomains- the N-terminal and C-terminal subdomains with GTP-binding and GTPase-activating sites, respectively. These are separated by a central core helix (the H7 helix) [6,7]. There is also a flexible C-terminal region which varies in length in different bacterial species [8]. Although FtsZ is a structural homologue of the eukaryotic cytoskeletal protein tubulin [9], it has distinctive structural differences to mammalian tubulin. This allows for FtsZ inhibitors with selective activity against bacterial cell division. One major structural difference is an interdomain cleft adjacent the H7-core helix that separates the two subdomains of FtsZ, a feature which is not present in tubulin [10]. Additionally, the linker that follows the C-terminal globular domain of FtsZ (also known as the disordered region) is thought to play a role in tethering and maintenance of protein–protein interactions that are essential for bacterial cell division [4].

**Figure 1:**
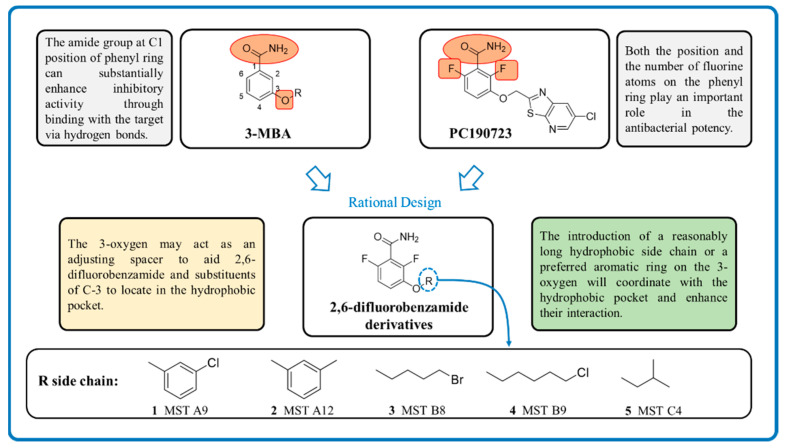
Design strategy of the novel 2,6-difluorobenzamide derivatives described previously for compounds MST A9 (**1**) [23], A12 (**2**) [23], B8 (**3**) [24], B9 (**4**) [24] and C4 (**5**) [24].

Various classes of small-molecule inhibitors of FtsZ have been identified such as derivatives of 3-methoxybenzamide (3-MBA, Figure 1), berberine derivatives, quinolinium compounds, polyphenolic derivatives, cinnamaldehyde, quinuclidines and quinazolines [4,11,12,13]. Among these inhibitors, PC190723 (a 2,6-difluoro derivative of benzamide with a thiazolopyridine moiety attached through an ether linker, Figure 1), has been one of the most extensively studied. PC190723 was shown to clear a *S. aureus* infection in a mouse bacteraemia model, thereby helping to establish FtsZ as an antibacterial drug target [14]. Crystallography studies have revealed that PC190723 binds into the narrow, hydrophobic pocket within the interdomain cleft of *S. aureus* FtsZ described above [7]. Structure-activity relationship (SAR) studies have indicated that the amide and difluoro groups at the 2- and 6-positions are important for activity [15,16,17]. The thiazolopyridine moiety of PC190723 forms interactions with the hydrophobic cleft between H7 helix and the C-terminal subdomain. It has also been shown that PC190723 and the 2,6-difluoro-3-methoxybenzamide fragment induce bundling of *S. aureus* and *Bacillus subtilis* FtsZ, acting as an FtsZ polymer stabilizer [18]. More recently, derivatives of PC190723 with improved solubility and pharmacokinetic properties have been developed, including the prodrug TXA709 which is currently in Phase 1 clinical development [19].

While various 2,6-difluorobenzamide derivatives including PC190723 and its prodrug have demonstrated excellent activity against Gram-positive bacteria including *S. aureus*, they have generally exhibited poor activity against Gram-negative species [20]. However, some studies have now demonstrated activity for selected 2,6-difluorobenzamide derivatives against Gram-negative species including *E. coli*, *Klebsiella pneumoniae* and *Acinetobacter baumannii* [20,21,22] when the resistance-nodulation-cell division (RND)-type efflux pumps in these species are either genetically or chemically inhibited. This indicates that these compounds are substrates of RND pumps, thus reducing their antimicrobial activity.

Previously our group has sought to further probe whether new 2,6-difluorobenzamide derivatives with other non-heterocyclic modifications to the 3-alkyloxy side chain could lead to increased antibacterial activity, broader spectrum of activity and improved on-target potency [23,24]. It was hypothesized that retaining the amide and 2,6-difluoro functions but introducing a series of small hydrophobic side chains such as substituted benzyl, alkyl halides, or a branched alkyl would enhance interactions with the hydrophobic pocket in the interdomain cleft of FtsZ. The design strategy to chemically optimize novel 2,6-difluorobenzamide derivatives is shown in Figure 1. Compounds, including representative MST compounds (**1**–**5**) showed encouraging antibacterial activity against the standard strains of *S. aureus* and *B. subtilis* [23,24].

In this report, we sought to further understand the antibacterial activity of 2,6-difluorobenzamide derivatives **1**–**5** against a broader spectrum of bacterial species, including ESKAPE pathogens [25]. We also undertook further detailed studies to understand the effects of these compounds in methicillin resistant *Staphylococcus aureus* (MRSA), a high priority pathogen for new antibiotic discovery and development [26]. These studies included tests for synergistic activity with the β-lactam oxacillin [27] in the standard strain and clinical isolates of MRSA, in vitro assays to confirm on-target effects of the compounds on FtsZ from MRSA, effects on mammalian tubulin and mammalian cell toxicity.

## 2. Results and Discussion

### 2.1. Antimicrobial Activity of MST Compounds

We have previously reported on the synthesis and antimicrobial activity of a series of 3-methoxybenzamide derivatives based on the 2,6-difluorobenzamide pharmacophore [23,24]. For the current study, five compounds (**1**–**5**) were selected based on their promising antimicrobial activity against β-lactamase producing *S. aureus* [28]. In the current study, these compounds were further screened for antimicrobial activity against an extended panel of Gram-positive (including MRSA ATCC 43300) and Gram-negative bacteria.

In agreement with our earlier reports, all the compounds displayed antimicrobial activity against methicillin sensitive *S. aureus* (MSSA ATCC25923, Table 1) with MIC values ranging from 1–8 µg/mL. Compound **4** was the most potent antibacterial with a low MIC of only 1 μg/mL. Additionally, all five compounds displayed excellent antimicrobial activity against the methicillin resistant *S. aureus* (MRSA ATCC 43300) with **2**, **4** and **5** displaying low MIC values of only 4 µg/mL against this highly drug resistant strain (Table 1). In order to further validate their anti-MRSA potential, the activities of **1**–**5** was also investigated upon an extended panel of 16 well-characterized clinical isolates (phenotypes and genotypes reported in Supplementary Table S2). The MST compounds inhibited growth of all clinical MRSA strains, confirming the MST compounds as potent antimicrobials against MRSA.

The FtsZ protein from *S. aureus* is unique in that it has a larger interdomain cleft than FtsZ from other bacteria, hence can accommodate more bulky inhibitors. This has been offered as an explanation for the selective activity of PC190723 against *S. aureus* and not against other Gram-positive pathogens. In comparison, the 3-*O*-alkyl functions of the tested MST compounds are smaller compared to that of PC190723 and could potentially fit the interdomain cleft of FtsZ from diverse Gram-positive bacteria. Accordingly, we also observed some modest antimicrobial activity against a vancomycin resistant *Enterococcus faecium* isolate for the 3-isopentyloxy derivative C4 (**5**) (Table 1).

The compounds were also tested for antimicrobial activity against a range of Gram-negative bacteria (Supplementary Table S1). None of the compounds were active against the standard and wild-type strains of the selected Gram-negative pathogens. Interestingly however, MST A12 (**2**), B8 (**3**) and B9 (**4**) displayed antibacterial activity against an *E. coli* strain lacking the AcrAB components of the AcrAB-TolC antibiotic efflux pump with MIC value of 128, 256 and 64 µg/mL respectively (Supplementary Table S1). This result indicates that antibiotic efflux pumps are partially responsible for the intrinsic resistance observed for Gram-negative organisms against the MST compounds. Similarly, it was also shown previously that TXA436, a prodrug of the difluorobenzamide PC190723, is a substrate for drug efflux pumps as addition of the efflux pump inhibitor phenylalanine-arginine β-naphthylamide (PAβN) rendered *E. coli* sensitive to this inhibitor [20].

We also examined the effect of the addition of colistin at sub-MIC concentration on the inhibitory effects of **1**–**5** on *E. coli* ATCC 25922 (Table 2). Colistin is a polymyxin antibiotic that, like other cationic lipopeptides, disrupts the outer membrane of Gram-negative bacteria increasing its permeabilization and permitting chemicals that are otherwise excluded to enter into the bacterial cells [27,29,30]. In the presence of colistin at 0.125 µg/mL (MIC 0.5 µg/mL), a reduction in the MIC was observed for three of the compounds with the greatest increase in activity noted with MST B9 (**4**) and MST C4 (**5**). This finding suggests that the lack of activity against Gram-negative bacteria may be at least partially explained by the inability of the compounds to breach the outer membrane permeability barrier.

### 2.2. MST Compounds Reverse Resistance to Oxacillin in MRSA ATCC 43300 and Clinical MRSA Isolates

**Figure 2:**
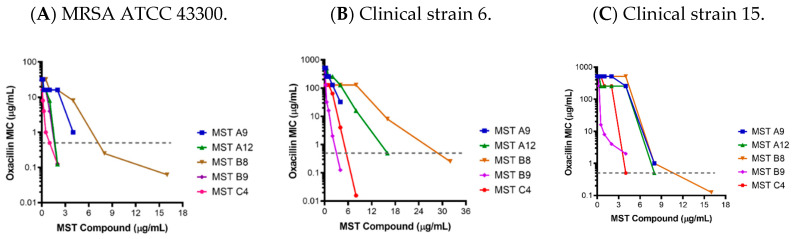
The MST compounds synergize with oxacillin to restore sensitivity in methicillin resistant *Staphylococcus aureus*. The MIC of MRSA ATCC 43300 and 16 clinical MRSA strains were determined in the presence of varying concentrations of the MST compounds. Results for the MRSA ATCC strain (**A**) and two representative clinical isolates (**B**,**C**) are shown with the graphs of all other clinical MRSA strains provided in Supplementary Figure S1. The MIC of MSSA ATCC 25923 for oxacillin (0.5 µg/mL) is indicated as a dotted grey line.

MRSA is resistant to almost all β-lactam antibiotics such as methicillin and oxacillin [28]. Since new peptidoglycan biosynthesis is synchronized with the formation of the Z-ring at mid-cell and constriction, we hypothesized that FtsZ inhibitors may synergize with cell-wall synthesis inhibitors, such as β-lactam antibiotics. In order to test this proposition, the MICs of oxacillin against a range of MRSA strains were determined in the presence of varying concentrations of the MST compounds using standard checkerboard assays [30]. In agreement with the hypothesis, all five compounds completely reversed resistance to oxacillin in MRSA ATCC 43300 with the MIC (32 µg/mL) decreasing to below that measured for the methicillin sensitive strain ATCC 25923 (0.5 µg/mL; Figure 2). Importantly, this reversal of resistance was universally observed with the panel of clinical MRSA strains (Figure 2 and Figure S1).

The most dramatic effects were observed with MST C4 (**5**) on clinical strain 15 where the MIC dropped by 1024-fold from 512 to 0.5 µg/mL (Figure 2). Synergism with methicillin or partial reversal of resistance against different MRSA ATCC strains were also observed in other studies for derivatives of benzofuroquinolinium, quinoline and TXA709 [12,13,14,28,31,32,33].

### 2.3. Time Kill Curves for MST Compounds Indicate Bactericidal or Bacteriostatic Mechanisms

**Figure 3:**
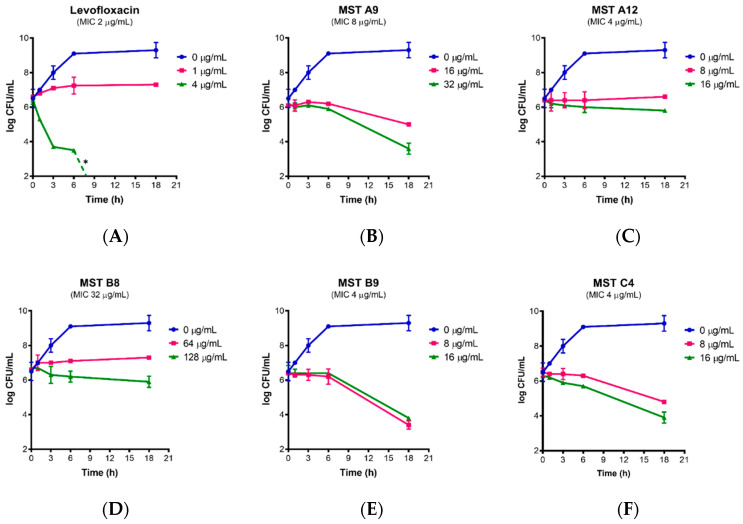
Time-kill curves for MST compounds. Viable cell counts were performed for (**A**) MRSA in the absence of any levofloxacin (blue line) and in the presence of levofloxacin at bacteriostatic (1 µg/mL) and bactericidal (4 µg/mL) concentrations; (**B**–**F**) in the presence of no compound (blue line), 2× MIC of the compounds (pink line) and 4× MIC of the compounds (green line). The results are representative of three independent experiments performed with different batches of cells and are presented as mean log CFU/mL ± SEM. The asterisk (*) represents viable cell counts below 1 × 10^3^ CFU/mL.

Bacterial survival assays were conducted to determine whether MST compounds were bactericidal or bacteriostatic in their action. Levofloxacin served as a control as this antibiotic is bacteriostatic at 0.5× MIC, but bactericidal at higher concentrations (Figure 3A). MRSA was incubated with the MST compounds at either 2× or 4× their MIC and viable cell counts performed at various time points from which time-kill curves were plotted (Figure 3B–F). In all cases, cell numbers remained constant for the first 6 h of treatment. Compounds MST A9 (**1**), B9 (**4**) and C4 (**5**) reduced the viable cell count after 6 h consistent with bactericidal activity (up to 3 log reductions). In contrast, cell numbers remained constant throughout the time course for MST A12 (**2**) and MST B8 (**3**) suggesting bacteriostatic activity. Previous studies have reported that other benzamide derivatives are bactericidal in their mode of action [4,11,12,34,35,36].

### 2.4. MST Compounds Disrupt Cellular Division

**Figure 4:**
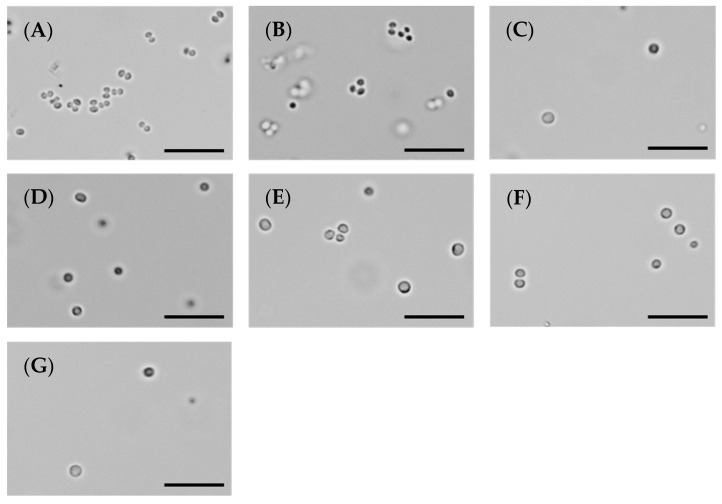
The MST compounds inhibit cell division in MRSA ATCC 43300. A culture of MRSA ATCC 43300 was adjusted to a density of approximately 1.5 × 10^8^ CFU/mL in MH broth and incubated at 37 °C. A 1 µL aliquot was taken out at varying timepoints (0, 1, 3, 6 and 18 h) and imaged by light microscope at a magnification of 100×. The images shown here were taken at 6 h post incubation with the MST compounds; a pre-bactericidal timepoint according to the kill kinetics from Figure 3. Images are (**A**) in the absence of the compounds, (**B**) with the divisome inhibitor, divin at 64 µg/mL, or with the MST compounds at 2× MIC values: (**C**) MST A9 (**1**) at 16 µg/mL, (**D**) MST A12 (**2**) at 8 µg/mL, (**E**) MST B8 (**3**) at 64 µg/mL, (**F**) MST B9 (**4**) at 8 µg/mL and (**G**) MST C4 (**5**) at 8 µg/mL. Scale bar is 50 µm.

To confirm that the antibacterial mechanism of the MST compounds was through the disruption of cellular division, microscopic analysis was performed. The morphology of MRSA ATCC 43300 treated with MST compounds at 2× and 4× their MIC values was observed using light microscopy at various time points (summarized in Figure 4 at 6 h treatment and expanded in Supplementary Figure S1). Bacteria treated with the known cell disruptor divin produced the ballooning phenotype that is characteristic of cocci that have enlarged but are unable to undergo division (Figure 4A vs. Figure 4B). The MST compounds induced the same enlarged phenotype for *S. aureus* within 3 h of exposure for the bactericidal compounds MST A9 (**1**), B9 (**4**) and C4 (**5**), which preceded the reduction in cell viability observed in the above time-kill study (Figure 3). The effect of 2× the MIC of MST compounds after 6 h of incubation are shown in Figure 4C–G. In the presence of the MST compounds, the *S. aureus* cells displayed a cell-division phenotype consistent with disrupted cell division, as evidenced by enlargement of or ballooning being observed (Figure 4 and Supplementary Figure S2) [34,36].

Benzamide derivatives has been shown to be effective inhibitors of cell division in *S. aureus* by our group [23,24] and others [34,36]. Inhibition of cellular division with a concomitant enlarged morphology (ballooning for cocci [37,38] and filamentation for bacilli [39,40] respectively) is one of the main features of FtsZ targeting compounds. This straightforward whole cell in vivo assay is often used as a screening tool to identify FtsZ inhibitors, however on-target effects by direct interaction with FtsZ need to be verified by confirming the effect of any putative inhibitors on purified protein in vitro.

### 2.5. Preparation of Recombinant FtsZ from Staphylococcus Aureus

In order to directly study the effect of the MST compounds on FtsZ from *S. aureus* (SaFtsZ), we prepared recombinant FtsZ for over-expression in *E. coli*. Genomic DNA was isolated from MRSA ATCC 43300 and the gene coding for FtsZ was amplified for expression in the pET-41a(+) vector. A C-terminal 8His-tag was added to allow purification with Ni-affinity chromatography. Conditions for culturing and induction of SaFtsZ in BL21(DE3) were optimized to produce a high level of over-expression of FtsZ that could be purified to homogeneity yielding about 20 mg/mL of pure SaFtsZ (42.38 kDa) per litre of culture (Supplementary Figure S3). This protein was subsequently employed for in vitro biochemical assays to characterise the activity of the MST compounds upon SaFtsZ. 

### 2.6. MST Compounds Enhance SaFtsZ GTPase Activity

**Figure 5:**
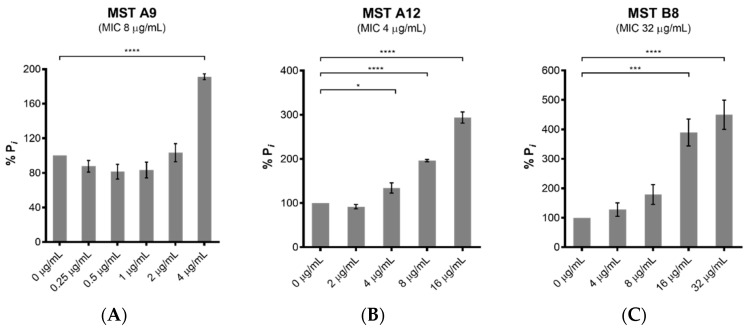

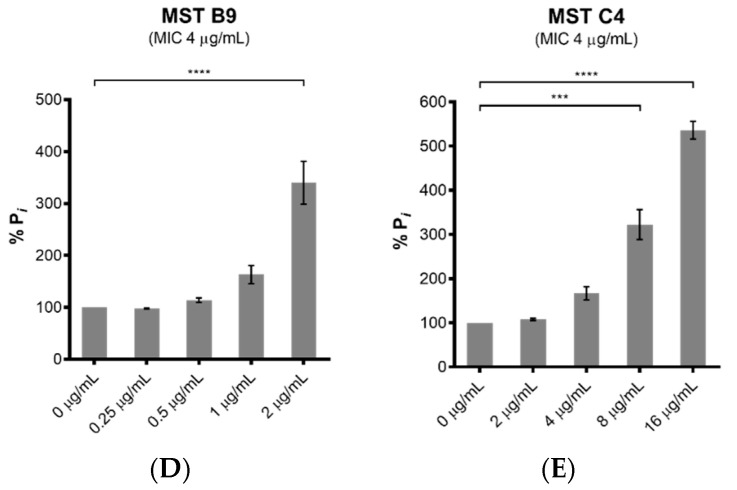
The MST compounds stimulate GTPase activity in a dose-dependent manner. The GTPase activity of purified SaFtsZ (23.6 nmol) was determined from the liberation of P*_i_* from GTP using malachite green. SaFtsZ was incubated with five of the MST compounds as indicated (**A**–**E**) for 30 min before the reaction was initiated by addition of 1 mM GTP and the reaction terminated by the addition of citric acid after 10 min. The P*_i_* release rate was normalized relative to the sample without the addition of MST compounds (0.514 mM Pi/mg SaFtsZ/min) that was taken as 100%. The results are presented as the mean ± SEM. Statistical analysis was performed using one-way ANOVA and statistical significance was represented with asterisks (*) as shown in the Figure (* *p* < 0.05; *** *p* < 0.005; **** *p* < 0.001).

The polymerization of FtsZ during cell division requires GTP. On-target activity was confirmed by measuring the effect of the MST compounds on the GTPase activity of purified SaFtsZ. All five MST compounds displayed a dose-dependent stimulation of SaFtsZ GTPase activity, with at least double the hydrolysis rate measured with the highest concentration of compound assayed in each case (Figure 5). Noteworthy was MST C4 (**5**) that increased SaFtsZ activity by 3-fold at 0.5× MIC and greater than 5-fold at the MIC. Similarly, Straniero et al., also reported a time dependence increase of GTPase activity with 2,6-difluorobenzamide on SaFtsZ that dropped off to baseline, unstimulated GTPase activity levels after 25 min [41].

### 2.7. MST Compounds Stabilize SaFtsZ Polymerization

**Figure 6:**
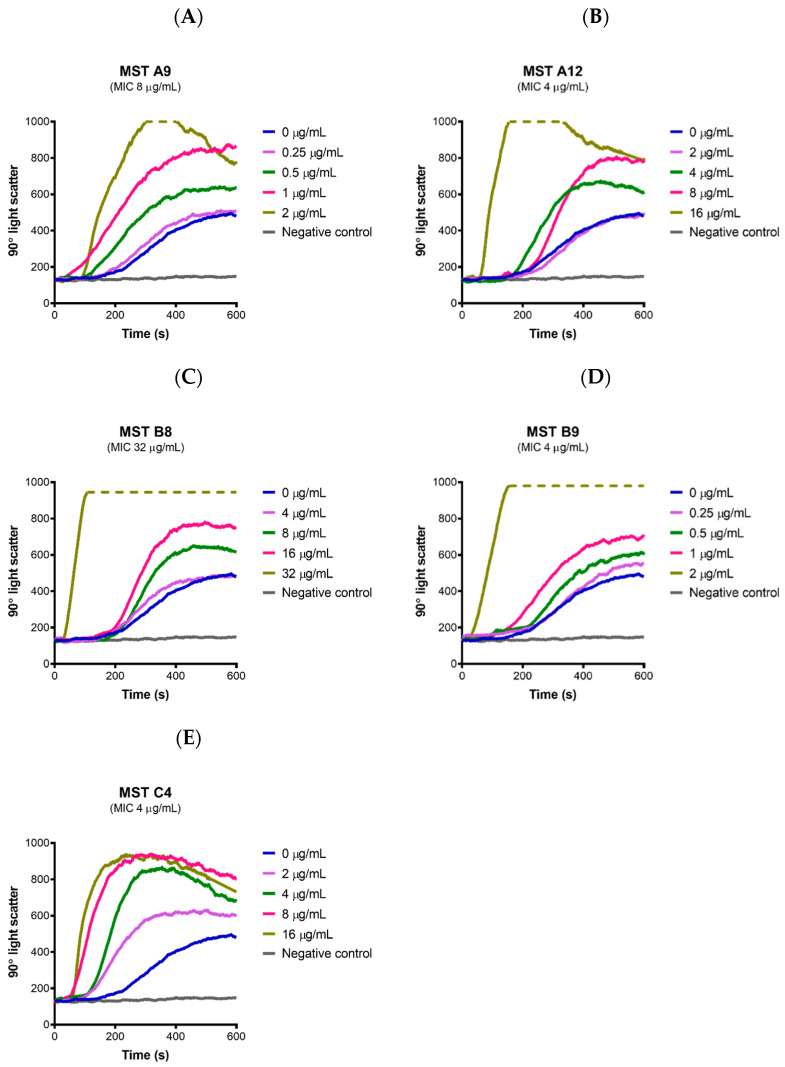
The MST compounds enhanced polymerization in SaFtsZ. (**A**–**E**) SaFtsZ (1 mg/mL) was pre-incubated in polymerization buffer (25 mM PIPES pH 6.8, 50 mM KCl and 10 mM MgCl_2_) together with the test compound indicated at the desired concentration for 300 s. Polymerization was then initiated with the addition of 1 mM GTP. The fluorescence was measured as a function of time at excitation and emission wavelengths of A_350_ and A_350_ nm, respectively, and a slit width of <2 nm. The temperature of the flow cell was kept constant at 20 °C for the duration of the experiment. The blue line in each panel indicates the addition of 2% (*v*/*v*) DMSO alone and coloured lines represent the various concentrations of compounds as indicated on the graph. The grey line represents a negative control where 1 mM GDP was added instead of GTP. Broken lines indicate that the light scatter signal was above the maximum detection limit of the fluorometer.

Since all the MST compounds affected the GTPase activity of SaFtsZ, the on-target effect of MST compounds on the dynamics of FtsZ polymerization was measured directly using 90° light scattering in a thermostatically controlled fluorescence spectrometer in which changes in FtsZ polymerization are reflected by corresponding changes in absorbance at A_350_ nm. A dose-dependent stimulation of the FtsZ polymerization was observed for all the compounds with an increase in both the steady state level of polymerization as well as the polymerization rate (Figure 6). Polymerization was specific to GTP as addition of GDP in the reaction resulted in no change to the reflected signal as expected if FtsZ retained its monomeric state. Together these data are consistent with the microscopy and biochemical data described above and confirm that the compounds stimulate FtsZ polymerization and prevent depolymerization during cell division. This overstimulation of FtsZ polymerization by FtsZ-targeting benzamides has been previously observed by us [24] and others [18,36,42,43,44]. The X-ray crystal structure of the benzamide inhibitor PC190723 bound to SaFtsZ reveals the binding site located in the interdomain cleft between the C-terminal domain and helix 7 thus stabilizing the protein in the high-affinity state necessary for protofilament assembly [42]. Biochemical analysis further revealed that PC190723 induced a dose-dependent decrease in the critical concentration for polymerization with concomitant increase in GTPase hydrolysis rate [42]. Similarly, the MST compounds caused a dose-dependent increase in GTPase rate (Figure 5) which increased the rate of polymerization (Figure 6) and decreased the critical concentration needed for assembly at constant temperature (results not shown). This mechanism of action is similar to that of the anticancer drug paclitaxel that stabilizes the polymers formed by eukaryotic tubulin [45]. This mechanism of action for the benzamides, including the MST compounds, is different from that of the quinoliniums [31,46] and tiplaxtinin [47] where a stabilization of polymerization of FtsZ was observed while the GTPase activity was reduced [4,11,12,13,48].

### 2.8. MST Compounds Do Not Affect the Polymerization of Mammalian Tubulin

**Figure 7:**
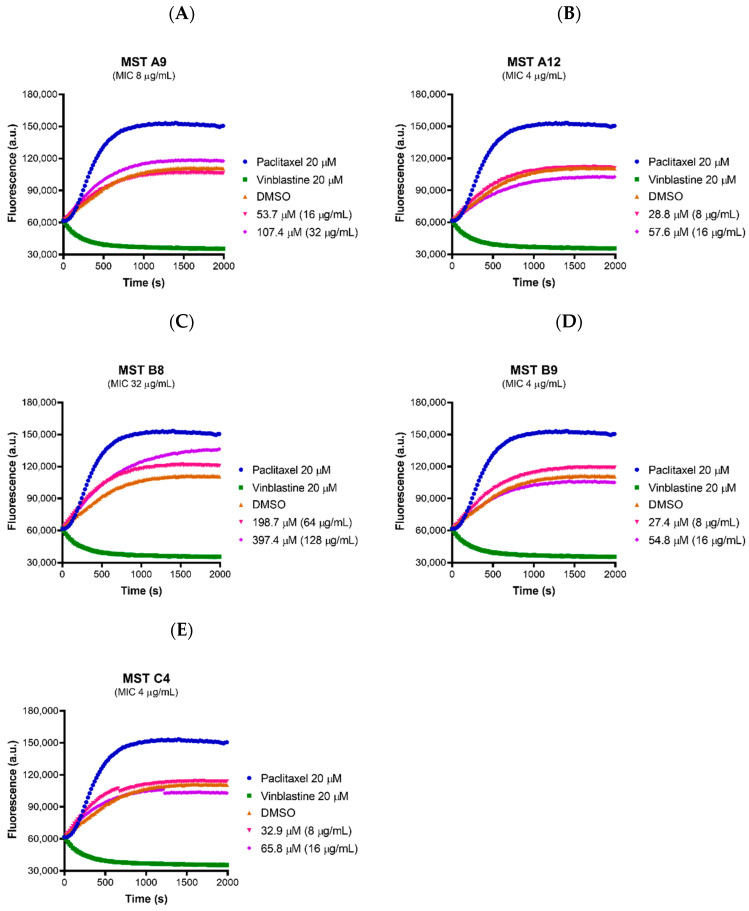
The MST compounds do not affect polymerization of mammalian tubulin. (**A**–**E**) The activity of the compounds on porcine tubulin was assessed using a commercial Tubulin Polymerization Assay Kit (Cytoskeleton, Inc.) following the protocol described by the manufacturer. The MST compounds at 2× and 4× MIC were pre-incubated with tubulin on ice before the reaction was initiated by the addition of 1 mM GTP. The fluorescence was measured as a function of time at A_360_ and A_420_ nm excitation and emission wave lengths, respectively, at the constant temperature of 37 °C. A 2% (*v*/*v*) DMSO (brown curves) vehicle control was used with paclitaxel (polymerization stabilizer; blue curves) and vinblastine (polymerization inhibitor; green curves) at 20 µM each also included as controls.

Tubulin is the closest mammalian homologue to bacterial FtsZ. Compounds designed to inhibit FtsZ but that show cross-reactivity to mammalian tubulin would be cytotoxic to mammalian cells. In order to verify that the compounds act selectively on bacterial FtsZ, the effect of all the compounds on the polymerization of mammalian tubulin was determined using a commercial tubulin assay kit. None of the compounds affected polymerization of mammalian tubulin at 2× and 4× their MIC values, with the exception of MST B8 (**3**) where a marginal increase in tubulin polymerization was observed at 4× MIC although the rate was much slower compared to paclitaxel (Figure 7). These data suggest the MST compounds confer the desired selective activity towards the bacterial FtsZ protein.

### 2.9. Toxicity of the MST Compounds

**Figure 8:**
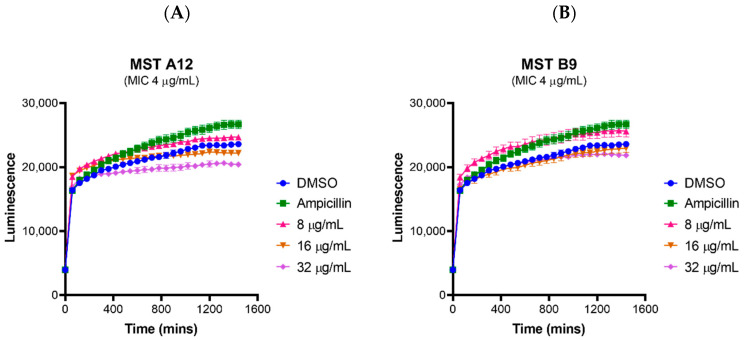
MST compounds are not cytotoxic to mammalian cells at concentrations of 2× MIC. Two compounds A12 (**A**) and B9 (**B**) are presented as examples to illustrate these effects. Real-time cell viability measurements for HepG2 after treatment with compounds at 2× (pink line), 4× (brown line) and 8× (purple line) MIC. Controls of 1% (*v*/*v*) DMSO (vehicle control, blue line) and 50 µg/mL ampicillin (green line) were used. Cell viability was measured every 5 min for 24 h at 37 °C and 5% CO_2_ on a Cytation5^®^ Cell Imaging Multi-Mode Reader (Bio-Tek^®^) using the RealTime-Glo^TM^ MT Cell Viability Assay reagent. The results are presented as the mean ± SEM (SEM is presented at every hour).

Although the MST compounds did not display any off-target activity on mammalian tubulin in a biochemical assay, we also wanted to rule out any non-specific cytotoxicity of the compounds against a mammalian cell line. The effect of the MST compounds on HepG2 ATCC HB-8065 mammalian liver cells was investigated using the RealTime-Glo^TM^ MT Cell Viability Assay Kit that is based on the ability of metabolically active cells to reduce the NanoLuc substrate which is then converted to luminescent signal. None of the compounds displayed any cytotoxicity at 2× their MIC, with MST A12 (**2**) and B9 (**4**) showing no cytotoxicity at 4× and 8× their MIC values (Figure 8). A marginal reduction of luminescence signal was observed at the high concentration of 8× the MIC for A9 (**1**) and A12 (**2**), while compounds B8 (**3**) and C4 (**5**) also displayed some cytotoxicity at the highest concentrations (Figure S4). Two compounds A12 (**2**) and B9 (**4**) are used as examples to illustrate these effects in Figure 8, and an expanded version showing the results of all compounds is provided in Supplementary Figure S4. The cytotoxicity was only observed at concentrations far exceeding those necessary for complete reversal of oxacillin resistance in a range of MRSA strains as indicated in Figure 2.

**Figure 9:**
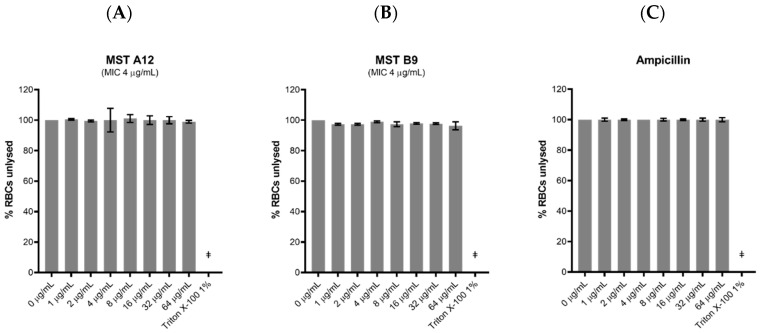
The MST compounds display no haemolytic activity. Freshly washed human RBCs in fresh PBS solution (137 mM NaCl, 2.7 mM KCl, 1.46 mM KH_2_PO_4_, 8.1 mM NaH_2_PO_4_, pH 7.4) were exposed to 2 µL MST compounds with concentrations ranging from 0 to 64 µg/mL in 1% (*v*/*v*) DMSO. A 1% (*v*/*v*) Triton X-100 solution was used to indicate complete RBC lysis (ǂ). Two compounds A12 (**A**) and B9 (**B**) are shown as examples to illustrate this effect. Ampicillin (0–64 µg/mL) was used as example of drug that does not cause RBC lysis (**C**). The assays were performed in quadruplicates. The plates were incubated at 37 °C while constantly shaking at 100 rpm for 1 h. Intact RBCs were removed by centrifugation and the presence of haemolytic products in the supernatant was determined by measuring the absorbance at A_450_ nm. The results are presented as the mean ± SEM. Statistical analysis was performed using a one-way ANOVA and indicated no statistically significant change in RBC lysis (*p* > 0.05).

Haemolytic activity of the MST compounds against human RBCs showed that none of the compounds displayed any haemolytic activity at concentrations up to 64 µg/mL (Figure 9 and Figure S5), a concentration that exceeds the antimicrobial concentration of the MST compounds by 2- to 16-fold. Two compounds A12 (**2**) and B9 (**4**), and a positive control ampicillin are used as examples to illustrate these effects in Figure 9, and the expanded version showing the results for all compounds is provided in Supplementary Figure S5.

**Figure 10:**
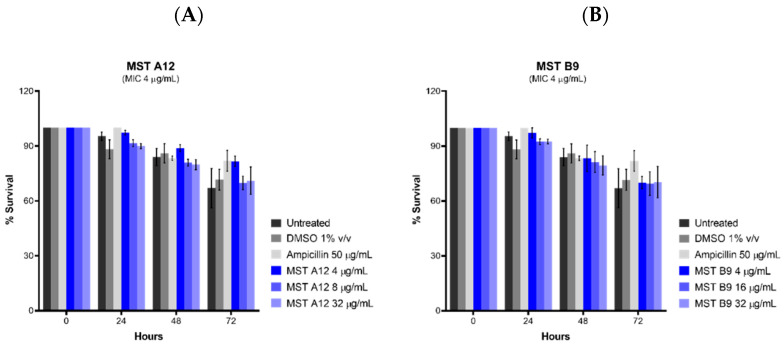
The MST compounds A12 (**A**) and B9 (**B**) did not display cytotoxicity in *Caenorhabditis elegans* nematodes. *C. elegans* nematodes were cultured on nematode growth media, with *E. coli* as its primary source of nutrient. Newly harvested nematodes were investigated for toxicity in the presence of the MST compounds at 2×, 4× and 8× their MIC values for a timespan of up to 72 h. The nematodes were counted under a light microscope at 400× magnification and the live nematodes at 72 h was indicated as a fraction of the starting number of nematodes (percentage survival). The results are presented as the mean ± SEM. Statistical analysis was performed using two-way ANOVA.

Toxicity of the MST compounds was also investigated against *Caenorhabditis elegans* nematodes in vivo in the presence of the MST compounds at 2×, 4× and 8× their MIC values. Live nematodes which can be distinguished from their physical appearance (refer Supplementary Figure S6) were counted every 24 h across 72 h under a light microscope at 400× magnification. Two compounds, A12 (**2**) and B9 (**4**) did not display any form of toxicity against *C. elegans* (Figure 10). Other compounds, A9 (**1**), B8 (**3**) and C4 (**5**) however, displayed some toxicity at the highest concentration (Supplementary Figure S7). This result is in agreement with that seen with cytotoxicity in mammalian liver cells (Figure 8).

## 3. Materials and Methods

### 3.1. Chemicals

All chemicals were from Sigma or Chem-Supply unless otherwise indicated.

### 3.2. Synthesis of Compounds

Derivatives of 2,6-difluorobenzamides **1** to **5** (Figure 1) were synthesized, chemically characterized and assessed for purity, according to the methods described previously [23,24]. All compounds were dissolved in DMSO. In all biological assays, a final DMSO concentration of 2% (*v*/*v*) was used unless otherwise specified.

### 3.3. Bacterial Strains and Growth Conditions

Reference bacterial strains were obtained from the ATCC. A series of clinical isolates of MRSA were kindly provided by Professor Geoffrey Coombs (Antimicrobial Resistance and Infectious Diseases Research Laboratory, Murdoch University, Perth, WA; PathWest Laboratory Medicine, Nedlands, WA, Australia). Details of these isolates have been previously published (Supplementary Table S2) [49]. Vancomycin resistant *Enterococcus faecium* (VRE 734, an isolate from wastewater from the Venter Laboratory Collection) was isolated from wastewater on Bile Aesculin agar (Oxoid, Australia) containing 6 µg/mL vancomycin and identified using MALDI-TOF mass spectrometry (Bruker, Preston, Victoria, Australia, Australian Centre for Antimicrobial Resistance Ecology). The *Pseudomonas aeruginosa* WT PAO1 and *Escherichia coli* with a deletion of both acrA and acrB drug efflux encoding genes were obtained from the Venter laboratory stock [50]. The protocol for microbial growth was adopted from the European Committee on Antimicrobial Susceptibility Testing (EUCAST) [51].

Non-fastidious bacteria and clinical isolates were maintained in Mueller Hinton (MH) broth (Acumedia, Lancashire, United Kingdom) supplemented with 15% (*v*/*v*) glycerol (Chem-Supply, Australia), cultured on Mueller Hinton (MH) agar (Acumedia, United Kingdom) plate and incubated at 37 °C. Fastidious bacteria were maintained in Brain Heart Infusion (BHI) broth (Acumedia, United Kingdom) supplemented with 15% (*v*/*v*) glycerol at −80 °C. It was cultured on MH agar plates supplemented with 5% (*v*/*v*) lysed horse blood and 3% (*v*/*v*) foetal bovine serum and incubated at 30 °C in the presence of 5% CO_2_. Drug susceptibility assays were carried out with the non-fastidious cultures grown in cation-adjusted Mueller Hinton (CaMH) broth (Becton Dickinson, Grenoble, France). *Streptococcus pyogenes* was grown in CaMH broth supplemented with 5% (*v*/*v*) lysed horse blood and 3% (*v*/*v*) foetal bovine serum whereas *Enterococcus faecium* was grown in BHI broth.

### 3.4. Drug Susceptibility Assay

The minimum inhibitory concentrations (MICs) for all five compounds were determined using the broth microdilution protocol as per EUCAST with an inoculum of approximately 1.6 × 10^6^ CFU/mL in an exponential growth phase [51]. Bacterial suspension, 100 µL/well, was added to 100 µL/well of drug dilutions, then incubated for 18 h at 37 °C. Fastidious bacterial suspensions were instead incubated at 30 °C in the presence of 5% CO_2_. Bacterial growth was assessed by measuring the change in A_600_ nm between time 0 and 18 h (Cytation5^®^ plate reader, Bio-Tek^®^). Levofloxacin, vancomycin and oxacillin were used as positive controls. Assays were completed in duplicate in at least three independent experiments.

Drug susceptibility assays were also performed on *E. coli* ATCC 25922 in the presence of a sub-MIC concentration of colistin (0.125 µg/mL). At this concentration colistin will permeabilize the outer membrane of E. coli, allowing compounds access to their target site.

### 3.5. Synergistic Activity between Oxacillin and Benzamide Derivatives on MRSA

To determine if the resistance displayed by MRSA strain towards β-lactam antibiotics could be reversed in combination with the compounds, interactions between the oxacillin and the compounds were assessed by a checkerboard titration assay.

Oxacillin was added in the first row and serially diluted in CaMH broth along the ordinate of the microwell plate. The MST compounds were then added and serially diluted along the abscissa. Finally, 100 µL/well of bacterial suspension (MRSA ATCC 43300 or clinical MRSA isolates) were added as described for the MIC assays. Bacterial growth was assessed by measuring the change in A_600_ nm between time 0 and 18 h (Cytation5^®^ plate reader, Bio-Tek^®^).

To evaluate the synergistic activity between the antibiotic and the compounds, we calculated the fractional inhibitory concentration index (FICI) according to the formula (Supplementary Table S3) [52];

(1) $$\mathrm{FICI}\;=\;\frac{{\mathrm{MIC}}_{\;\mathrm{antibiotic}\;\mathrm{in}\;\mathrm{combination}\;\mathrm{with}\;\mathrm{compound}}}{{\mathrm{MIC}}_{\;\mathrm{antibiotic}\;\mathrm{only}}}\;+\;\frac{{\mathrm{MIC}}_{\;\mathrm{compound}\;\mathrm{in}\;\mathrm{combination}\;\mathrm{with}\;\mathrm{antibiotic}}}{{\mathrm{MIC}}_{\;\mathrm{compound}\;\mathrm{only}}}$$

For the compounds to synergize with the antibiotic, the FICI must be ≤ 0.5 [52]. The FICI values for all the MST compounds are provided in Supplementary Table S4.

### 3.6. Time Kill Assays against MRSA

To assess growth inhibition kinetics, and to determine bactericidal or bacteriostatic effects, the compounds were tested at 2× MIC and 4× MIC against MRSA ATCC 43300. The bacterial suspension of approximately 2.7 × 10^8^ CFU/mL was prepared in a 1.5 mL Eppendorf^®^ tube. An aliquot was then serially diluted into a microtiter plate before being spot plated onto a fresh MH agar plate. The tube was incubated, and the process was repeated at 1, 3, 6 and 18 h post-exposure to the compounds. All MH agar plates were incubated at 37 °C for 18 h. Colonies were counted to calculate the CFU/mL. Dilutions were prepared to ensure the count was kept between 2 to 20 colonies per spot. Levofloxacin was used as a control at sub-MIC (1 µg/mL) and 2× MIC (4 µg/mL) concentrations to produce bacteriostatic and bactericidal effects, respectively. The results from each drug concentration were obtained from at least three independent experiments with different batches of cells.

### 3.7. Determination of Cell Division Phenotype

Morphological changes of bacteria when exposed to the compounds were assessed microscopically. Eppendorf^®^ tubes were set up with MRSA ATCC 43300 as described for the time-kill assays. At 0, 1, 3, 6 and 18 h, an aliquot of the bacterial suspension (1 µL) was transferred onto a glass slide and viewed under a light microscope (Olympus CX33^®^) at 100× magnification. Representative images were captured using JENOPTIK GRYPHAX^®^ imaging.

### 3.8. Cloning of MRSA ATCC 43300 FtsZ Protein

SaFtsZ was obtained via PCR amplification from genomic DNA of MRSA ATCC 43300 using forward 5′-AGGGTTTCATATGTTAGAATTTGAACAAGGATTTAATC-3′ and reverse 5′-AGGGTTTCTCGAGACGTCTTGGTTCTTCTTGAACG-3′ primers containing NdeI and XhoI sites respectively (underlined). The amplicon was then ligated into the NdeI and XhoI sites of the pET-41a(+) (Novagen) vector. The resulting 8-His tagged FtsZ construct was confirmed by Sanger sequencing. This plasmid was transformed into *E. coli* DH5α^TM^ silver (Bioline) for amplification then subsequently into *E. coli* BL21(DE3) for recombinant production.

### 3.9. Over-Expression and Purification of FtsZ Protein

Bacterial culture was grown in prewarmed Luria Bertani (Becton Dickinson, France) broth at 37 °C for 5 h before cooling to 25 °C. SaFtsZ expression was subsequently induced by the addition of 1 mM isopropyl -β-D-1-thiogalactopyronoside (IPTG; Thermo-Fisher, Australia) for 18 h at 25 °C. The cells were harvested by centrifugation at 5000× *g* before suspension in Buffer A (50 mM Tris HCl pH 8.0, 200 mM NaCl and 10% (*w*/*v*) glycerol) supplemented with cOmplete^TM^, EDTA-free Protease Inhibitor Cocktail (Roche) and 20 µg/mL DNAse (Sigma, Australia) and lysed using a cell disruptor (Constant Systems E1061, Thermo Scientific, Australia) at 30 kPsi. The lysate was clarified by ultra-centrifugation (Optima XPN-100 Ultracentrifuge, Beckman Coulter, Australia) at 200,000× *g* for 45 min. SaFtsZ was then purified from the supernatant using 2 × 1 mL HisTrap HP columns (GE Healthcare) on an Äkta FPLC (GE Healthcare). Protein solution was loaded onto the column pre-equilibrated in Buffer A, washed for 10 column volumes with Buffer A containing 20 mM imidazole then eluted with Buffer A in a gradient of 100 to 150 mM imidazole. As SaFtsZ is devoid of aromatic amino acids, and consequently does not absorb UV light, fractions from the protein purification containing the desired protein were identified by SDS-PAGE (4–12% NuPAGE Bis-Tris polyacrylamide gels, (Invitrogen, Australia)). Fractions containing purified SaFtsZ were pooled and exchanged into Buffer A using a HiTrap desalting column (GE Healthcare) to remove imidazole. Aliquots of the purified FtsZ were snap frozen in liquid nitrogen and stored at −80 °C.

### 3.10. Protein Concentration Determination

The protein concentration was measured using the BioRad^TM^ BCA Protein Assay Standard Kit, according to the manufacturer’s instructions with bovine serum albumin used as a standard. The A_750_ nm was measured using a Cytation5^®^ plate reader (Bio-Tek^®^).

### 3.11. GTPase Assay

Effects of the compounds on the GTPase activity of SaFtsZ were assessed using a malachite green-phosphomolybdate colorimetric assay with alterations to the methods previously described [53]. A Reactive Enzymatic Media (REM solution) was prepared by adding malachite green from a 1% *v*/*v* stock solution (Thermo Scientific, Australia) to 9 mM ammonium molybdate dissolved in 1 M hydrochloric acid to give a final malachite green concentration of 0.0453% (*v*/*v*). The REM solution was filtered through a 0.45 µm micropore filter (MiniSart, Sartorius Stedim Biotech, GmBH Germany), and stored at 4 °C for no more than 5 days. The REM solution was activated using Triton X-100 10% (*v*/*v*) in a ratio of 1:100, kept on ice, protected from light and used within 24 h. 

The GTPase assay was carried out using three 96-microwell plates (Corning Costar, China). In the reaction plate, SaFtsZ (1 mg/mL) was mixed with the compounds at a defined concentration in GTP reaction buffer (50 mM Tris HCl pH 7.2, 300 mM KCl and 5 mM MgCl_2_). Solvent control wells received an appropriate concentration of DMSO 2% (*v*/*v*). The reaction was initiated with the addition of 1 mM GTP, and a negative control with the addition of 1 mM GDP.

Upon initiation of the reaction, 10 µL of the mix was transferred to the analytical plate that had been pre-filled with 50 µL/well of activated REM solution. The reaction was ceased 1 min thereafter, by adding 25 µL/well of 34% (*w*/*v*) citric acid.

The reaction plate was kept in the dark and incubated at 37 °C, constantly shaken at 120 rpm for 15 min before repeating the procedure on a separate analytical plate. The analytical plate was incubated in the dark at 37 °C for 30 min, then A_600_ nm was measured using a PerkinElmer Enspire® plate reader. A standard curve of known phosphate concentrations; KH_2_PO_4_ (Chem-Supply, Australia) at concentrations 0, 25, 50, 100, 200, 300 and 400 µM was included in each experiment to quantify the extent of GTP hydrolysis.

### 3.12. Effects on SaFtsZ Polymerization Using 90° Light Scattering

A reaction mix was prepared in a cold Eppendorf® tube where SaFtsZ (1 mg/mL) was mixed with the test compound at the desired concentration and a polymerization buffer diluted from a double strength buffer to give a final concentration of 25 mM PIPES pH 6.8, 50 mM KCl and 10 mM MgCl_2_. The reaction mix was prepared such that the DMSO concentration remained constant at 2% (*v*/*v*). The mix was then added into a quartz cuvette (PerkinElmer®, United Kingdom), and placed in a PerkinElmer® LS 55 fluorometer under the fixed condition of excitation/emission at 350/350 nm, slit width <2 nm, a 1 s read interval. The temperature was calibrated to remain constant at 20 °C. The fluorescence was followed for 300 s to allow equilibration, then polymerization was initiated with the addition of 1 mM GTP. A separate negative control was prepared where 1 mM GDP was added instead of the GTP. The reaction was followed for 600 s.

### 3.13. Mammalian Tubulin Polymerization Assay

The effect of the compounds on mammalian porcine tubulin was assessed using a Tubulin Polymerization Assay Kit (Cytoskeleton, Inc.; BK011P, Denver, CO, USA), following the protocol described by the manufacturer.

The MST compounds were tested at 2× MIC and 4× MIC. A final concentration of 2% (*v*/*v*) DMSO was maintained in the assay. Controls included 20 µM paclitaxel (promoter of polymerization), 20 µM vinblastine (inhibitor of polymerization) and 2% (*v*/*v*) DMSO (solvent control). The reaction was initiated by the addition of 1 mM GTP and the fluorescence was read for 2000 s using a PerkinElmer Enspire® plate reader.

### 3.14. Cytotoxicity Analysis of MST Compounds

In vitro cytotoxicity was assessed in HepG2 (ATCC HB-8065) cells using the RealTime-Glo^TM^ MT Cell Viability Assay Kit (Promega) essentially as described previously [54]. The MST compounds were tested at 2×, 4× and 8× MIC using a final DMSO concentration of 1% (*v*/*v*) in the assay. Two controls, 1% (*v*/*v*) DMSO and 50 µg/mL ampicillin were used. The luminescence signal was read at 5-min intervals over 24 h in a Cytation5^®^ plate reader (Bio-Tek^®^), at 37 °C in the presence of 5% CO_2_.

The haemolysis assay was performed using fresh human red blood cells (RBCs). PBS solution (137 mM NaCl, 2.7 mM KCl, 1.46 mM KH_2_PO_4_, 8.1 mM NaH_2_PO_4_ pH 7.4) was used to wash the RBCs three times at 500 g for 5 min, then they were resuspended in 1% (*w*/*v*) PBS solution. Compounds (2 µL) were added to a 96-microwell plate, and serially diluted from 64 µg/mL to 1 µg/mL in 1% (*v*/*v*) DMSO. Controls of 1% (*v*/*v*) Triton X-100, 1% (*v*/*v*) DMSO and 128 µg/mL ampicillin were used. These were performed in quadruplicates.

Thereafter, 198 µL RBCs were added into all wells and the plates were incubated at 37 °C under constant shaking (100 rpm) for 1 h. RBCs were precipitated by centrifugation of the plates (1000× *g* for 3 min). An aliquot (100 µL) of each supernatant was transferred into a new 96-microwell plate and the A_450_ nm was determined using a PerkinElmer Enspire^®^ plate reader. The fraction of intact RBC for each sample was determined as fraction of the intact RBCs for the sample without the addition of compounds (set at 100%) and plotted as a function of compound concentration.

The protocol in growing, hatching and harvesting of *Caenorhabditis elegans* nematodes were described [55]. The nematodes were grown on a nematode growth media (1 mM CaCl_2_, 1 mM MgSO_4_, 25 mM KPO_4_ and 5 mg/mL cholesterol) mixed with super-optimal agar (0.3% (*v*/*v*) NaCl, 1.7% (*w*/*v*) technical agar and 0.25% (*w*/*v*) peptone). Live infant nematodes were ‘chunk’ from an agar plate filled with an even lawn of *E. coli*. A small colony of the nematodes were visually inspected under the light microscope to ensure proper growth, and adult nematodes were harvested on day three.

Nematodes at a density of approximately 25 to 30 units/25 µL were transferred into a 96-microwell plate (25 µL/well) in a buffered optimized growth media (95% (*v*/*v*) M9 buffer, 5% (*w*/*v*) BHI media and 10 µg/mL cholesterol). The number of live nematodes were counted (t = 0 h) under a light microscope at 400× magnification. The MST compounds (**1** to **5**) at 2×, 4× and 8× their MIC values were added into the wells with at least 3 replicates using a final DMSO concentration of 1% (*v*/*v*). The plate was incubated at 25 °C and the number of live vs dead nematodes was counted every 24 h for 72 h. Separate wells containing 50 µg/mL ampicillin and 1% (*v*/*v*) DMSO were used as controls. The percentage of living nematodes at each time point across 72 h was calculated to give the percentage of survival.

## 4. Conclusions

This study was undertaken to explore in-depth antimicrobial activity and on-target effects of selected 2,6-difluorobenzamide derivatives with non-heterocyclic substituents attached through the 3-oxygen, that had previously been shown to inhibit standard strains of Gram-positive bacteria. When tested against high priority pathogens, the compounds exhibited antimicrobial activity against MRSA ATCC 4300 and highly resistant clinical strains of MRSA, with an isopentyloxy-substituted compound MST C4 (**5**) also displaying some activity against VRE. At sub-MIC concentrations, all of the compounds were able to reverse resistance to oxacillin in the clinical MRSA strains. This is an important observation as the β-lactams are one of the most widely prescribed classes of antibiotic. Cloning and expression of the MRSA ATCC 4300 FtsZ protein allowed in vitro characterization of the mechanisms of action of these compounds. We conclusively demonstrated that the compounds **1**–**5** specifically targeted SaFtsZ, causing a dose-dependent increase in GTPase rate, with an increased rate of polymerization and stabilization of the FtsZ polymers. At antimicrobial concentrations, the compounds did not affect mammalian tubulin and did not show haemolytic or cytotoxic activity in human cells or in an *in vivo C. elegans* cytotoxicity model, with two compounds, the 3-methylbenzyl derivatives MST A12 (**2**) and the chlorohexyl derivative B9 (**4**) showing no cytotoxicity at 4× and 8× their MIC values. Compound B9 (**4**) was the most potent and superior among all five compounds and should be further developed as the lead compound.

The compounds lacked activity against the tested Gram-negative pathogens. Interestingly however, in accordance with other recent studies [20,21,22], compounds MST A12 (**2**), B8 (**3**) and B9 (**4**) displayed some antibacterial activity against an *E. coli* strain lacking the AcrAB components of the AcrAB-TolC RND type drug efflux pump. Further, we showed that the MIC of some of these compounds in a standard strain of *E. coli* could be reduced in the presence of a sub-MIC concentration of the permeabilization enhancer colistin. Together these results suggest that the lack of activity against Gram-negatives can at least partly be attributed to their inability to reach inhibitory concentrations inside Gram-negative cells. This opens the possibility of further studies examining the antimicrobial activity of these compounds in the presence of efflux pump inhibitors, as well as their effects on the FtsZ protein of Gram-negative species including *E. coli* and *A. baumannii*.

The five 2,6-difluorobenzamide derivatives examined in this study are therefore excellent compounds for further development as antimicrobial agents or as resistance breakers to re-sensitize MRSA against a range of β-lactam antibiotics. Further, the frequency of resistance can be ascertained in future studies alongside the newly remodelled compounds.

## Figures and Tables

**Table 1:**
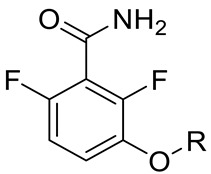
Minimum inhibitory concentration (MIC) of the MST compounds on *S. aureus* and *E. faecium*.

Compounds and Antimicrobial Agents		Minimum Inhibitory Concentration (MIC), µg/mL																		
		ATCC Strains		Clinical MRSA Strains																VRE WW 734
Compound	R side chain	MSSAATCC 25923	MRSAATCC 43300	C1	C2	C3	C6	C7	C8	C9	C10	C11	C13	C14	C15	C16	C18	C19	C20	
**1** MST A9	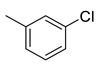	4	8	16	16	16	32	16	8	8	8	8	16	16	16	16	16	16	16	>64
3-((3-chlorobenzyl)oxy)-2,6-difluorobenzamide																				
**2** MST A12	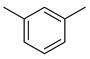	4	4	16	16	16	16	16	16	16	16	32	16	16	16	16	16	16	16	>64
3-((3-methylbenzyl)oxy)-2,6-difluorobenzamide																				
**3** MST B8	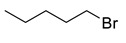	8	32	32	32	32	32	32	32	32	64	64	32	32	32	32	32	32	32	>64
3-((5-bromopentyl)oxy)-2,6-difluorobenzamide																				
**4** MST B9	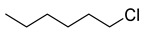	1	4	8	16	8	4	4	8	4	4	8	4	8	8	8	8	8	4	>64
3-((6-chlorohexyl)oxy)-2,6-difluorobenzamide																				
**5** MST C4	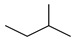	2	4	8	8	8	8	8	16	8	16	16	8	16	8	8	16	8	8	64
3-(isopentyloxy)-2,6-difluorobenzamide																				
Divin		>128	>128	n.t.	n.t.	n.t.	n.t.	n.t.	n.t.	n.t.	n.t.	n.t.	n.t.	n.t.	n.t.	n.t.	n.t.	n.t.	n.t.	n.t.
Oxacillin		0.5	32	64	64	32	512	256	32	256	256	512	128	512	512	32	64	64	32	>512
Levofloxacin		0.5	2	0.25	64	0.25	32	16	0.25	32	16	8	16	8	0.25	16	0.25	0.5	0.25	>64
Vancomycin		n.t.	0.5	1	1	1	1	0.5	1	0.5	1	1	1	1	4	1	1	1	1	64

Abbreviations: MSSA: methicillin sensitive *S. aureus*; MRSA: methicillin resistant *S. aureus;* VRE: vancomycin resistant *E. faecium;* n.t.: not tested.

**Table 2:** Change in the MIC of MST compounds for *E. coli* ATCC 25922 in the presence of colistin.

Compounds	MIC, µg/mL	
	Colistin	
	+0 µg/mL	+0.125 µg/mL
**1** MST A9	>256	>256
**2** MST A12	>256	128
**3** MST B8	>256	>256
**4** MST B9	>256	64
**5** MST C4	>256	64

## Supplementary Materials

The following are available online at https://www.mdpi.com/2079-6382/9/12/873/s1. Figure S1. The MST compounds synergizes with oxacillin to restore sensitivity in methicillin resistant Staphylococcus aureus. Figure S2. MST compounds were tested at 2× and 4× their inhibitory concentrations to determine phenotypic changes in MRSA. Figure S3. Purification of SaFtsZ. Figure S4. MST compounds are not cytotoxic to mammalian cells at concentrations of 2× MIC. Figure S5. The MST compounds display no haemolytic activity. Figure S6. *Caenorhabditis elegans* nematodes (no treatment) viewed under the light microscope at time 0 h (left) and 72 h (right). Figure S7. The MST compounds did not display cytotoxicity in *C. elegans* nematodes up at 2× their MIC values. Table S1: Antibacterial activity of the MST compounds on ESKAPE pathogens and other microorganisms tested in this study. Table S2. MRSA clone/isolate name, type, source, multi-locus, sequence type (MLST), staphylococcal cassette chromosome (SCCmec) type, clonal complex, Panton-Valentine leukocidin status (PVL) and spa type for isolates used in this study. Table S3. Criteria used for interpretation of the FICI obtained from checkerboard assays. Table S4. The FICI, calculated to three significant figures for each individual compound is tabulated below.
