# Supplementary material for: Antimicrobial Action and Reversal of Resistance in MRSA by Difluorobenzamide Derivatives Targeted at FtsZ
Source: Antibiotics (Basel). 2020 Dec 5;9(12):873. doi: 10.3390/antibiotics9120873 (PMC7762090; doi:10.3390/antibiotics9120873)
Supplement: Supplementary file 1 [file antibiotics-09-00873-s001.pdf]

antibiotics

Supplementary Materials

Antimicrobial Action and Reversal of Resistance in MRSA by Difluorobenzamide Derivatives Targeted at FtsZ

Wern Chern Chai [1], Jonathan J Whittall [1], Di Song [2], Steven W Polyak [1], Abiodun D Ogunniyi [3], Yinhu Wang [2,4], Fangchao Bi [2], Shutao Ma [2], Susan J Semple [1,5] and Henrietta Venter [1,*]

1 Health and Biomedical Innovation, Clinical and Health Sciences, University of South Australia, 5000 Adelaide, South Australia, Australia; wern_chern.chai@mymail.unisa.edu.au (W.C.C.); Jon.Whittall@unisa.edu.au (J.J.W.); Steven.Polyak@unisa.edu.au (S.W.P.); Susan.Semple@unisa.edu.au (S.J.S.); rietie.venter@unisa.edu.au (H.V.)
2 Department of Medicinal Chemistry, Key Laboratory of Chemical Biology (Ministry of Education), School of Pharmaceutical Sciences, Cheeloo College of Medicine, Shandong University, Jinan 250012, China; sondy321@163.com (D.S.); wangyinhu@lcu.edu.cn (Y.W.); yahe1111@163.com (F.B.); mashutao@sdu.edu.cn (S.M.)
3 Australia Centre for Antimicrobial Resistance Ecology, School of Animal and Veterinary Sciences, University of Adelaide, Roseworthy Campus, 5371 Roseworthy, Australia; david.ogunniyi@adelaide.edu.au
4 School of Pharmacy, Liaocheng University, Liaocheng 25200, China
5 Quality Use of Medicines and Pharmacy Research Centre, Clinical and Health Sciences, University of South Australia, 5000 Adelaide, South Australia, Australia
* Correspondence: rietie.venter@unisa.edu.au

antibiotics

MDPI

Supplementary Materials:

Table S1. Antibacterial activity of the MST compounds on ESKAPE pathogens and other microorganisms tested in this study.

**Strains**	LEF	OXA	MST compounds				
			A9	A12	B8	B9	C4
Acinetobacter baumannii ATCC 19606	0.5	>64	128	>256	>256	>256	>256
Enterobacter aerogenes ATCC 13408	1	>64	>256	>256	>256	>256	>256
Escherichia coli WT BW 25113	0.25	>64	>256	>256	>256	>256	>256
Escherichia coli BW ΔAcrAB	0.008	1	>256	128	256	64	>256
Klebsiella pneumoniae ATCC 4352	1	>64	>256	>256	>256	>256	>256
Klebsiella pneumoniae ATCC 13883	1	>64	>256	>256	>256	>256	>256
Klebsiella pneumoniae ATCC 33495	1	>64	>256	>256	>256	>256	>256
Pseudomonas aeruginosa WT PAO1	0.5	>64	>256	>256	>256	>256	>256
Pseudomonas aeruginosa ATCC 27853	1	>64	>256	>256	>256	>256	>256
Streptococcus pneumoniae (type 3, mucoid strain) ATCC 6303	1	n.t.	>256	>256	>256	>256	>256
Streptococcus pyogenes ATCC 10389	1	n.t.	>256	>256	>256	>256	>256

LEF = levofloxacin, OXA = oxacillin, n.t. = not tested

antibiotics

Table S2. MRSA clone/isolate name, type, source, multi-locus, sequence type (MLST), staphylococcal cassette chromosome (SCCmec) type, clonal complex, Panton-Valentine leukocidin status (PVL) and *spa* type for isolates used in this study.

No.	Name/Clone	Type	MLST	SCCmec	Clonal complex	PVL	*spa*	Number
1	WA1	CA-MRSA	ST1	Iva	1	NEG	t127	WBG 8287
2	WA2	CA-MRSA	ST78	Iva	88	NEG	t186	03-16926
3	WA3	CA-MRSA	ST5	Iva	5	NEG	t002	WBG 8378
4	WA84	CA-MRSA	ST45	V	45	NEG	t1081	07-16502
5	QLD PVL+	CA-MRSA	ST93	IVA	Singleton	POS	t202	03-16790
6	AUS2 EMRSA	HA-MRSA	ST239					
7	AUS3 EMRSA	HA-MRSA	ST239					
8	Classic MRSA	HA-MRSA	ST250	I	8	NEG		03-17590
9	Bengal Bay PVL+	CA-MRSA	ST772	V	1	NEG	t3387	07-17048
10	Irish EMRSA-1	HA-MRSA	ST8	II	8	NEG	tST498	
11	Irish EMRSA-2	HA-MRSA	ST8					
12	UK 15	HA-MRSA	ST22	IV	22	NEG	t022	
13	UK 15 PVL+	HA-MRSA	ST22	IVb	22	POS	t891	
14	UK 16 EMRSA	HA-MRSA	ST36	II	30	NEG	t081	
15	UK 17	HA-MRSA	ST247	I	8	NEG	t051	
16	Taiwan cMRSA	CA-MRSA	ST59	5(C2&5)	59	POS	t437	
17	New York/Japan	HA-MRSA	ST5	II	5	NEG	t242	03-16981
18	WS PP MRSA	CA-MRSA	ST30	Iva	30	NEG	t5074	08-19231
19	ST 398-MRSA-V	CA-MRSA	ST398	V	398	NEG	t034	09-16670
20	USA 300	CA-MRSA	ST8	IVc	8	POS	t008	04-15086

Coombs GW, Pearson J, Christiansen K, Nimmo GR. *Staphylococcus aureus* Programme 2010 (SAP 2010) Community Survey: MRSA Epidemiology and Typing Report.

Note: clinical strain 4, 5, 12 and 17 were excluded from this study as they demonstrated sensitivity towards oxacillin (MIC ≤ 8 μg/mL).

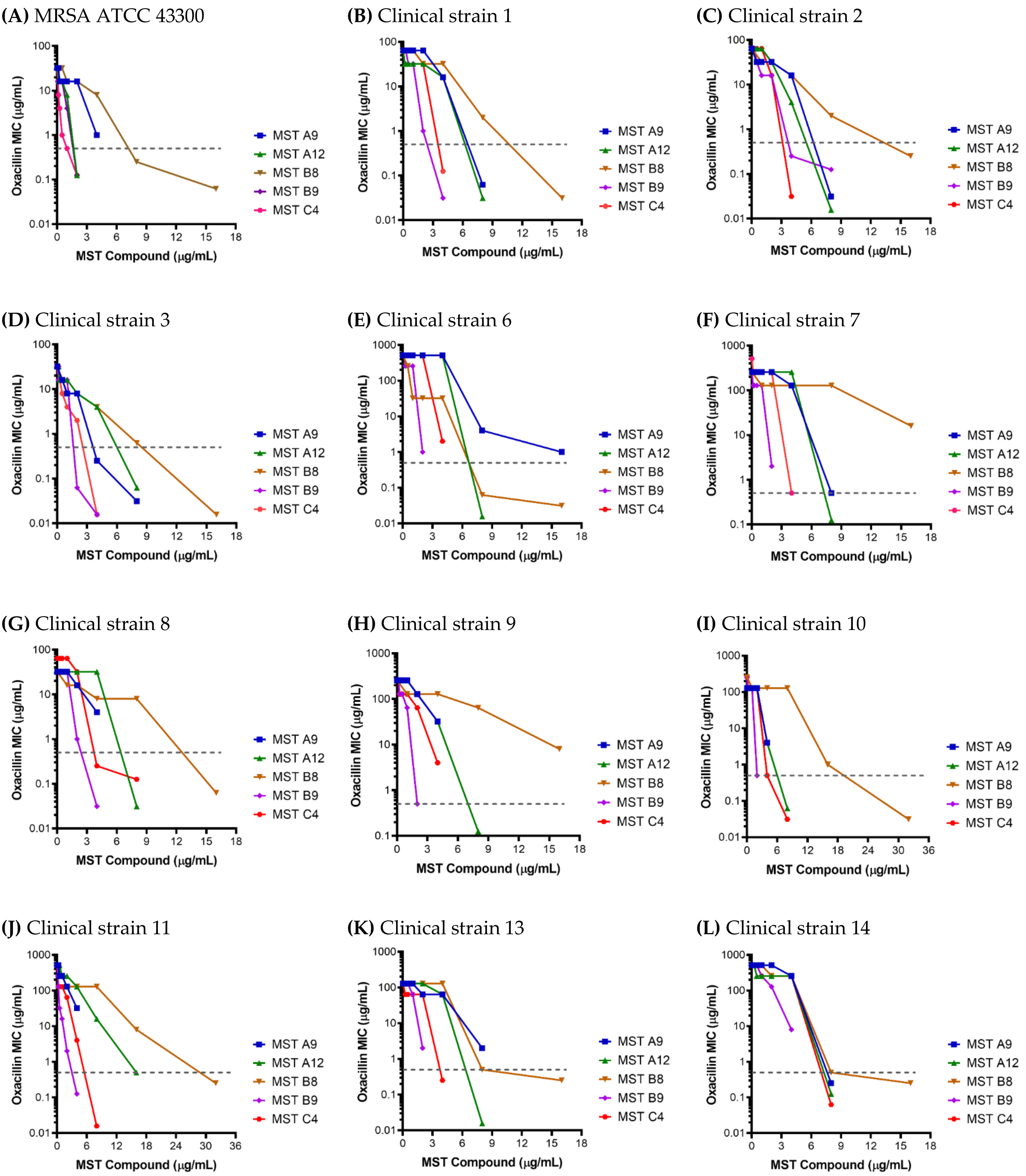

(A) MRSA ATCC 43300
(B) Clinical strain 1
(C) Clinical strain 2
(D) Clinical strain 3
(E) Clinical strain 6
(F) Clinical strain 7
(G) Clinical strain 8
(H) Clinical strain 9
(I) Clinical strain 10
(J) Clinical strain 11
(K) Clinical strain 13
(L) Clinical strain 14
Oxacillin MIC (µg/mL)
MST Compound (µg/mL)
MST A9
MST A12
MST B8
MST B9
MST C4

(M) Clinical strain 15

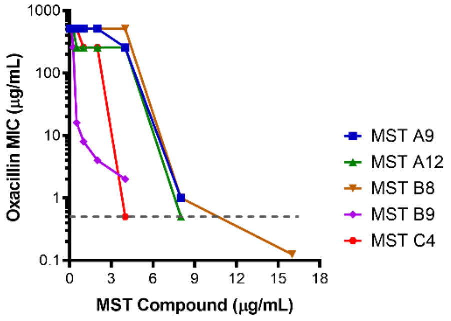

(N) Clinical strain 16

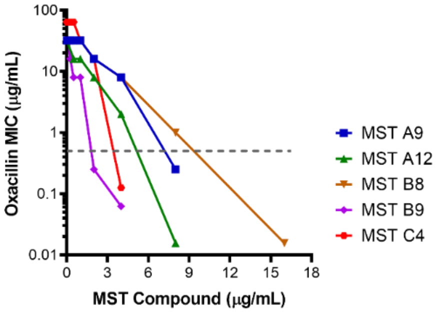

(O) Clinical strain 18

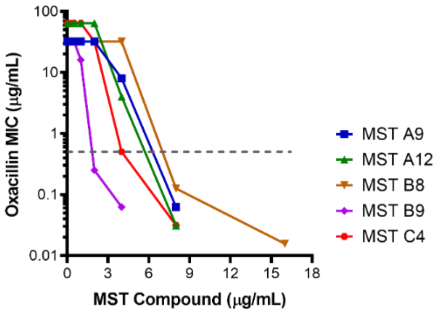

(P) Clinical strain 19

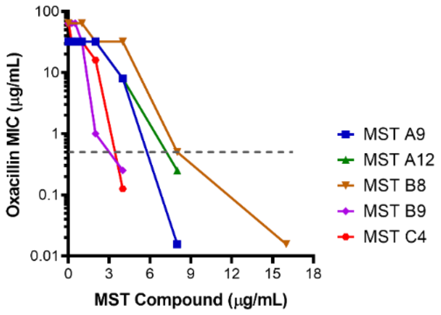

(Q) Clinical strain 20

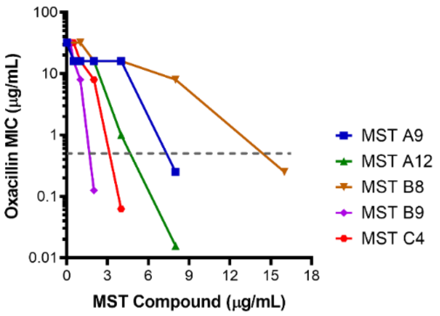

Figure S1. The MST compounds synergize with oxacillin to restore sensitivity in (A) methicillin resistant *Staphylococcus aureus* ATCC 43300 and (B-Q) 16 clinical MRSA strains

The MIC of (A) MRSA ATCC 43300 and (B-Q) 16 clinical MRSA strains were determined in the presence of varying concentrations of the MST compounds. The MIC of MSSA ATCC 25923 for oxacillin (0.5 µg/mL) is indicated in the grey dotted line.

To evaluate the synergistic activity between oxacillin and the compounds, the fractional inhibitory concentration index (FICI) was calculated using the formula below:

$$\text{FICI} = \frac{\text{MIC}_{\text{antibiotic in combination with compound}}}{\text{MIC}_{\text{antibiotic only}}} + \frac{\text{MIC}_{\text{compound in combination with antibiotic}}}{\text{MIC}_{\text{compound only}}}$$

For the compounds to synergize the activity of oxacillin, the FICI must be ≤ 0.5.

Table S3. Criteria used for interpretation of the FICI obtained from checkerboard assays.

FICI	Criteria
≤ 0.5	Synergistic
> 0.5 but < 1	Additive
≥ 1 but < 4	Indifferent
≥ 4	Antagonistic

This assay aimed to determine if the compounds could reverse the resistance of clinical MRSA isolates towards oxacillin. Two compounds demonstrated additive effect on two different clinical MRSA isolates- MST A9 on clinical MRSA isolate 10 and MST C4 on clinical MRSA isolate 6. However, a 32- and 256-fold reduction in oxacillin's MIC was observed (Table S4) with a reduction from 128 μg/mL to 4 μg/mL, and 512 μg/mL to 2 μg/mL, respectively. This suggests the compounds were equally efficacious in reversing the resistance of clinical MRSA isolate towards oxacillin.

antibiotics

MDPI

Table S4. The FICI, calculated to three significant figures for each individual compound is tabulated below.

MST A9

Strains	MIC (µg/mL)				Fold reduction of oxacillin MIC	FICI	Comments
	MST A9		Oxacillin				
	- Oxacillin	+ Oxacillin	- MST A9	+ MST A9			
MRSA ATCC 43300	16	0.25	32	1	32	0.063	Synergistic
Clinical Isolate 1	16	4	64	0.0625	1024	0.251	Synergistic
Clinical Isolate 2	16	0.5	64	0.03125	2048	0.032	Synergistic
Clinical Isolate 3	16	0.5	32	0.03125	1024	0.032	Synergistic
Clinical Isolate 6	32	8	512	1	512	0.252	Synergistic
Clinical Isolate 7	16	4	256	0.5	512	0.252	Synergistic
Clinical Isolate 8	8	2	32	4	8	0.375	Synergistic
Clinical Isolate 9	8	2	256	32	8	0.375	Synergistic
Clinical Isolate 10	8	4	128	4	32	0.531	Additive
Clinical Isolate 11	8	0.5	512	32	16	0.125	Synergistic
Clinical Isolate 13	16	2	128	2	64	0.141	Synergistic
Clinical Isolate 14	16	4	512	0.25	2048	0.250	Synergistic
Clinical Isolate 15	16	4	512	1	512	0.252	Synergistic
Clinical Isolate 16	16	2	32	0.25	128	0.133	Synergistic
Clinical Isolate 18	16	4	32	0.0625	512	0.252	Synergistic
Clinical Isolate 19	16	4	32	0.01563	2048	0.250	Synergistic
Clinical Isolate 20	16	0.5	32	0.25	128	0.039	Synergistic

MST A12

Strains	MIC (µg/mL)				Fold reduction of oxacillin MIC	FICI	Comments
	MST A12		Oxacillin				
	- Oxacillin	+ Oxacillin	- MST A12	+ MST A12			
MRSA ATCC 43300	4	0.125	32	0.125	256	0.035	Synergistic
Clinical Isolate 1	16	0.125	64	0.03125	2048	0.008	Synergistic
Clinical Isolate 2	16	2	64	0.01563	4096	0.125	Synergistic
Clinical Isolate 3	16	0.25	32	0.0625	512	0.018	Synergistic
Clinical Isolate 6	16	8	512	0.01563	32768	0.500	Synergistic
Clinical Isolate 7	16	8	256	0.125	2048	0.500	Synergistic
Clinical Isolate 8	16	4	32	0.03125	1024	0.251	Synergistic
Clinical Isolate 9	16	2	256	0.125	2048	0.125	Synergistic
Clinical Isolate 10	16	4	128	0.0625	2048	0.250	Synergistic
Clinical Isolate 11	32	1	512	0.5	1024	0.032	Synergistic
Clinical Isolate 13	16	4	128	0.01563	8192	0.250	Synergistic
Clinical Isolate 14	16	0.5	512	0.125	4096	0.031	Synergistic
Clinical Isolate 15	16	0.5	512	0.5	1024	0.032	Synergistic
Clinical Isolate 16	16	0.5	32	0.01563	2048	0.032	Synergistic
Clinical Isolate 18	16	4	64	0.03125	2048	0.250	Synergistic
Clinical Isolate 19	16	4	32	0.25	128	0.252	Synergistic
Clinical Isolate 20	16	0.5	32	0.01563	2048	0.032	Synergistic

MST B8

Strains	MIC (µg/mL)				Fold reduction of oxacillin MIC	FICI	Comments
	MST B8		Oxacillin				
	- Oxacillin	+ Oxacillin	- MST B8	+ MST B8			
MRSA ATCC 43300	32	1	32	0.0625	512	0.033	Synergistic
Clinical Isolate 1	32	2	64	0.03125	2048	0.063	Synergistic
Clinical Isolate 2	32	1	64	0.25	256	0.035	Synergistic
Clinical Isolate 3	32	1	32	0.01563	2048	0.032	Synergistic
Clinical Isolate 6	32	0.5	512	0.03125	16384	0.016	Synergistic
Clinical Isolate 7	32	1	256	16	16	0.094	Synergistic
Clinical Isolate 8	32	1	32	0.0625	512	0.033	Synergistic
Clinical Isolate 9	32	1	256	8	32	0.063	Synergistic
Clinical Isolate 10	64	1	256	0.03125	8192	0.016	Synergistic
Clinical Isolate 11	64	1	512	0.25	2048	0.016	Synergistic
Clinical Isolate 13	32	8	128	0.25	512	0.252	Synergistic
Clinical Isolate 14	32	2	512	0.25	2048	0.063	Synergistic
Clinical Isolate 15	32	8	512	0.125	4096	0.250	Synergistic
Clinical Isolate 16	32	2	32	0.01563	2048	0.063	Synergistic
Clinical Isolate 18	32	8	64	0.01563	2048	0.250	Synergistic
Clinical Isolate 19	32	2	64	0.01563	4096	0.063	Synergistic
Clinical Isolate 20	32	2	32	0.25	128	0.070	Synergistic

MST B9

Strains	MIC (µg/mL)				Fold reduction of oxacillin MIC	FICI	Comments
	MST B9		Oxacillin				
	- Oxacillin	+ Oxacillin	- MST B9	+ MST B9			
MRSA ATCC 43300	4	0.125	32	0.125	256	0.035	Synergistic
Clinical Isolate 1	8	0.5	64	0.03125	1024	0.063	Synergistic
Clinical Isolate 2	16	0.5	64	0.125	512	0.018	Synergistic
Clinical Isolate 3	8	0.125	32	0.0625	512	0.018	Synergistic
Clinical Isolate 6	4	0.25	512	1	512	0.064	Synergistic
Clinical Isolate 7	4	0.125	256	2	128	0.039	Synergistic
Clinical Isolate 8	8	2	32	0.03125	1024	0.251	Synergistic
Clinical Isolate 9	4	0.25	256	0.5	512	0.064	Synergistic
Clinical Isolate 10	4	0.125	256	0.5	512	0.033	Synergistic
Clinical Isolate 11	8	0.125	512	0.125	4096	0.016	Synergistic
Clinical Isolate 13	4	1	128	2	64	0.266	Synergistic
Clinical Isolate 14	8	1	512	8	64	0.141	Synergistic
Clinical Isolate 15	8	0.25	512	2	256	0.035	Synergistic
Clinical Isolate 16	8	0.25	32	0.0625	512	0.064	Synergistic
Clinical Isolate 18	8	1	32	0.0625	512	0.127	Synergistic
Clinical Isolate 19	8	1	64	0.25	256	0.129	Synergistic
Clinical Isolate 20	4	0.5	32	0.125	256	0.129	Synergistic

MST C4

Strains	MIC (µg/mL)				Fold reduction of oxacillin MIC	FICI	Comments
	MST C4		Oxacillin				
	- Oxacillin	+ Oxacillin	- MST C4	+ MST C4			
MRSA ATCC 43300	4	0.0625	32	0.25	128	0.023	Synergistic
Clinical Isolate 1	8	2	64	0.125	512	0.252	Synergistic
Clinical Isolate 2	8	2	64	0.03125	2048	0.250	Synergistic
Clinical Isolate 3	8	0.25	32	0.01563	2048	0.032	Synergistic
Clinical Isolate 6	8	4	512	2	256	0.504	Additive
Clinical Isolate 7	8	0.125	512	0.5	1024	0.017	Synergistic
Clinical Isolate 8	16	2	64	0.125	512	0.127	Synergistic
Clinical Isolate 9	8	0.25	256	4	64	0.047	Synergistic
Clinical Isolate 10	16	0.25	256	0.03125	8192	0.016	Synergistic
Clinical Isolate 11	16	0.25	512	0.01563	32768	0.016	Synergistic
Clinical Isolate 13	8	0.25	128	0.25	512	0.033	Synergistic
Clinical Isolate 14	16	1	512	0.0625	8192	0.031	Synergistic
Clinical Isolate 15	8	1	1024	1	1024	0.126	Synergistic
Clinical Isolate 16	8	1	64	0.125	512	0.127	Synergistic
Clinical Isolate 18	16	2	64	0.03125	1024	0.126	Synergistic
Clinical Isolate 19	8	0.25	64	0.125	512	0.033	Synergistic
Clinical Isolate 20	8	1	32	0.0625	512	0.127	Synergistic

antibiotics

MST compounds inhibit cellular division

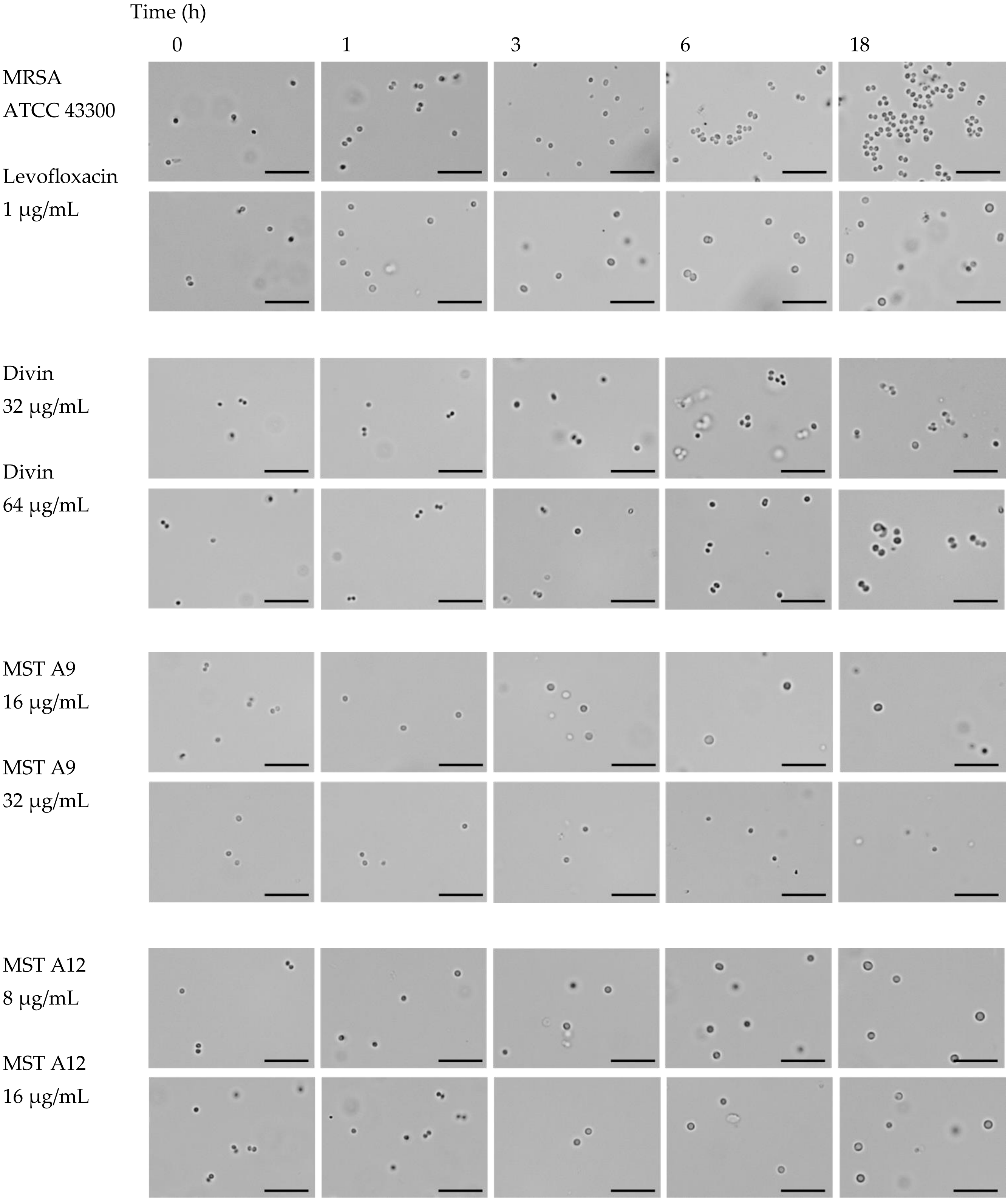

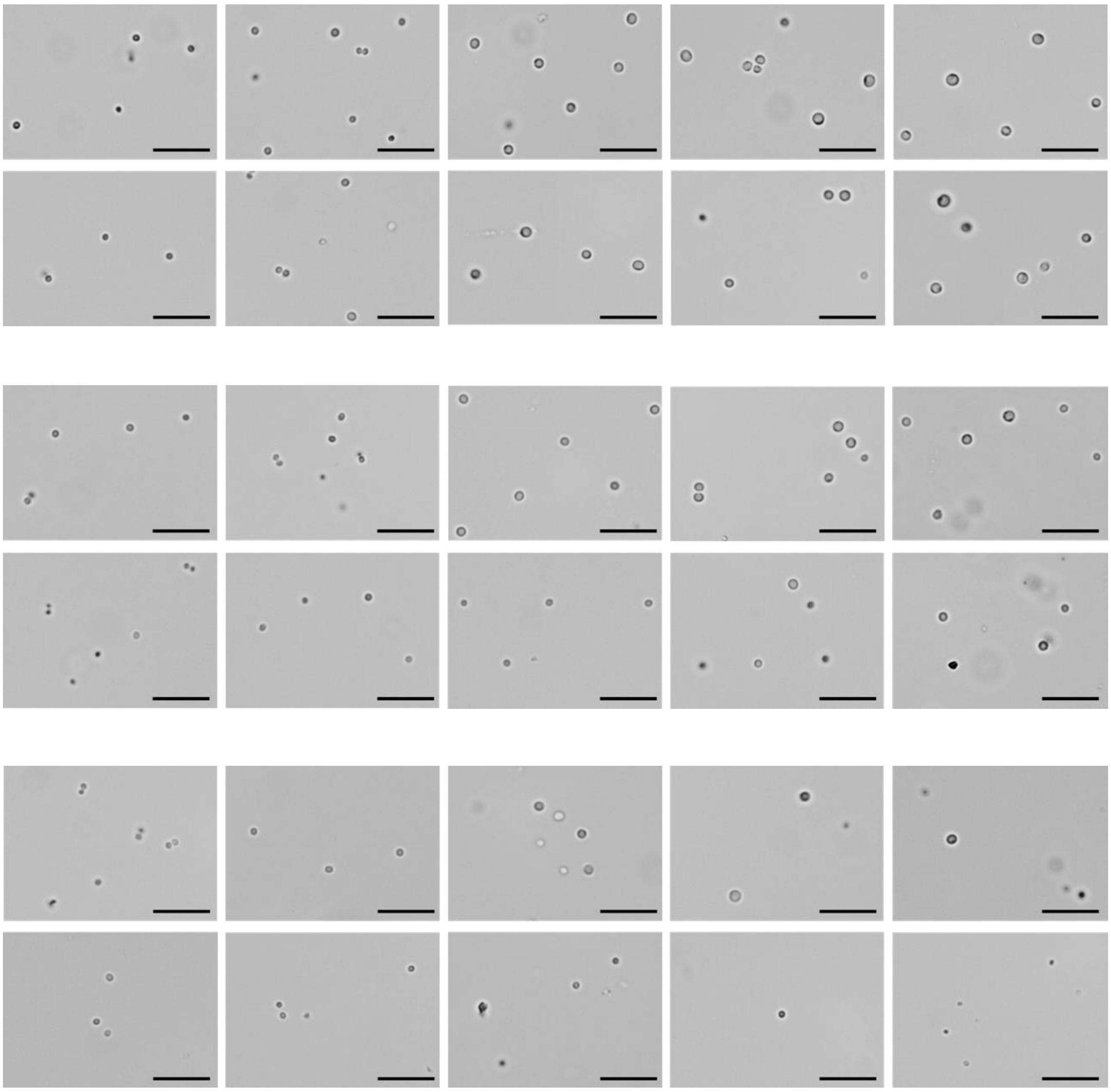

Figure S2. MST compounds were tested at 2× and 4× their inhibitory concentrations to determine phenotypic changes in MRSA.

The phenotypic changes in MRSA morphology was analysed under a light microscope (100× magnification) at time 0, 1, 3, 6 and 18 h. Scale bar is 50 µm.

Preparation of recombinant FtsZ from *S. aureus*

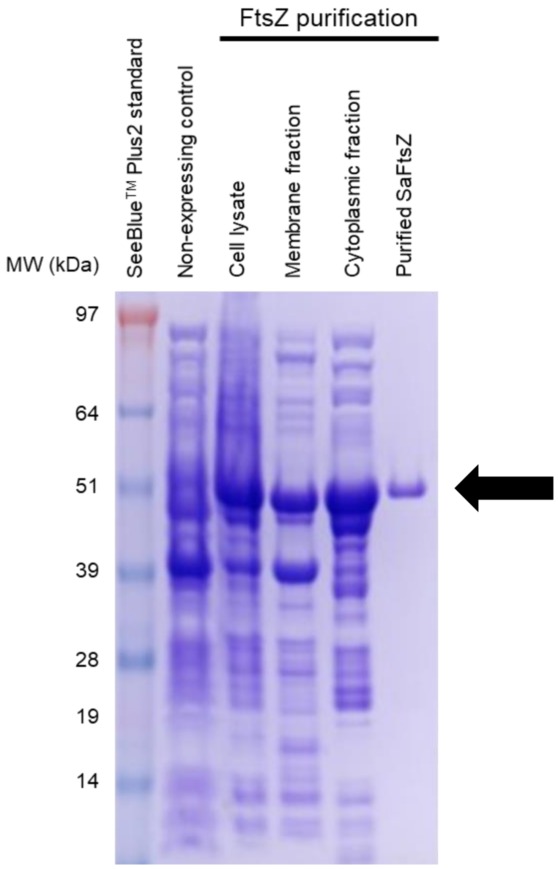

Figure S3. Purification of SaFtsZ.

SaFtsZ was cloned in the pET41a(+) vector and expressed in BL21(DE3) *E. coli* via induction with 1 mM IPTG. The protein was purified from the cytoplasmic fraction using Nickel-affinity column chromatography. The different fractions from the purification process were loaded onto SDS-PAGE (4-12% NuPAGE Bis-Tris polyacrylamide gel, Invitrogen Australia). Protein was visualized by staining with Coomassie® Brilliant Blue R-250 (BioRad™, Australia). Purified SaFtsZ is observed at about 50 kDa as indicated by the arrow. The protein concentration was determined using the standard BioRad™ BCA Protein Assay Kit.

Mammalian Cytotoxicity of the MST Compounds

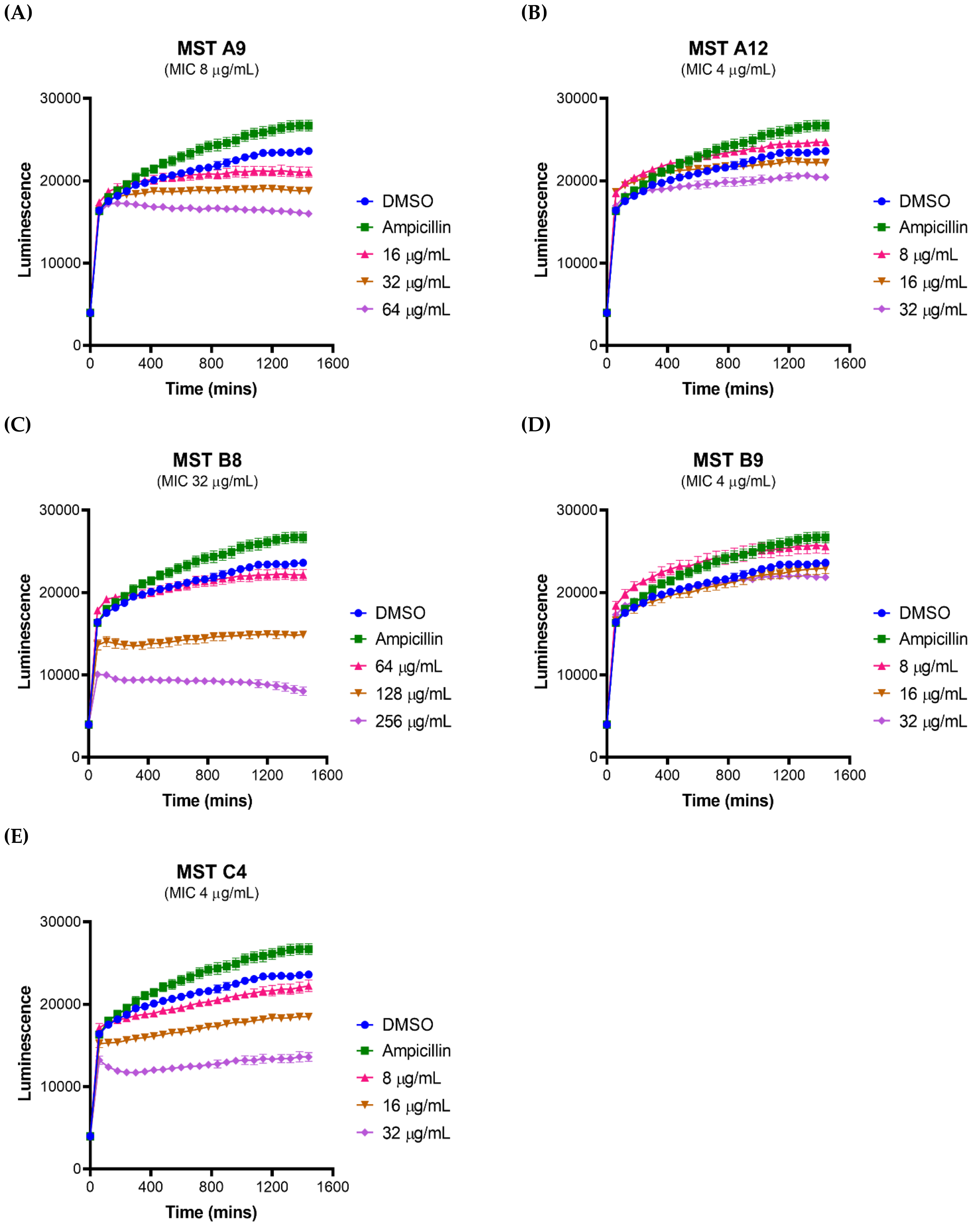

Figure S4. The MST compounds (A-E) are not cytotoxic to mammalian cells at concentrations of 2× MIC. Real-time cell viability measurements for HepG2 after treatment with 2× (pink line), 4× (brown line) and 8× (purple line) MIC values. A 1% (v/v) DMSO (vehicle control, blue line) and 50 µg/mL ampicillin (green line) were used as controls. Cell viability was measured every 5 minutes for 24 hours at 37 °C and 5% CO_2 on a Cytation5® Cell

Imaging Multi-Mode Reader (Bio-Tek®) using the RealTime-Glo™ MT Cell Viability Assay reagent. The results are presented in mean ± SEM (SEM was presented at every hour).

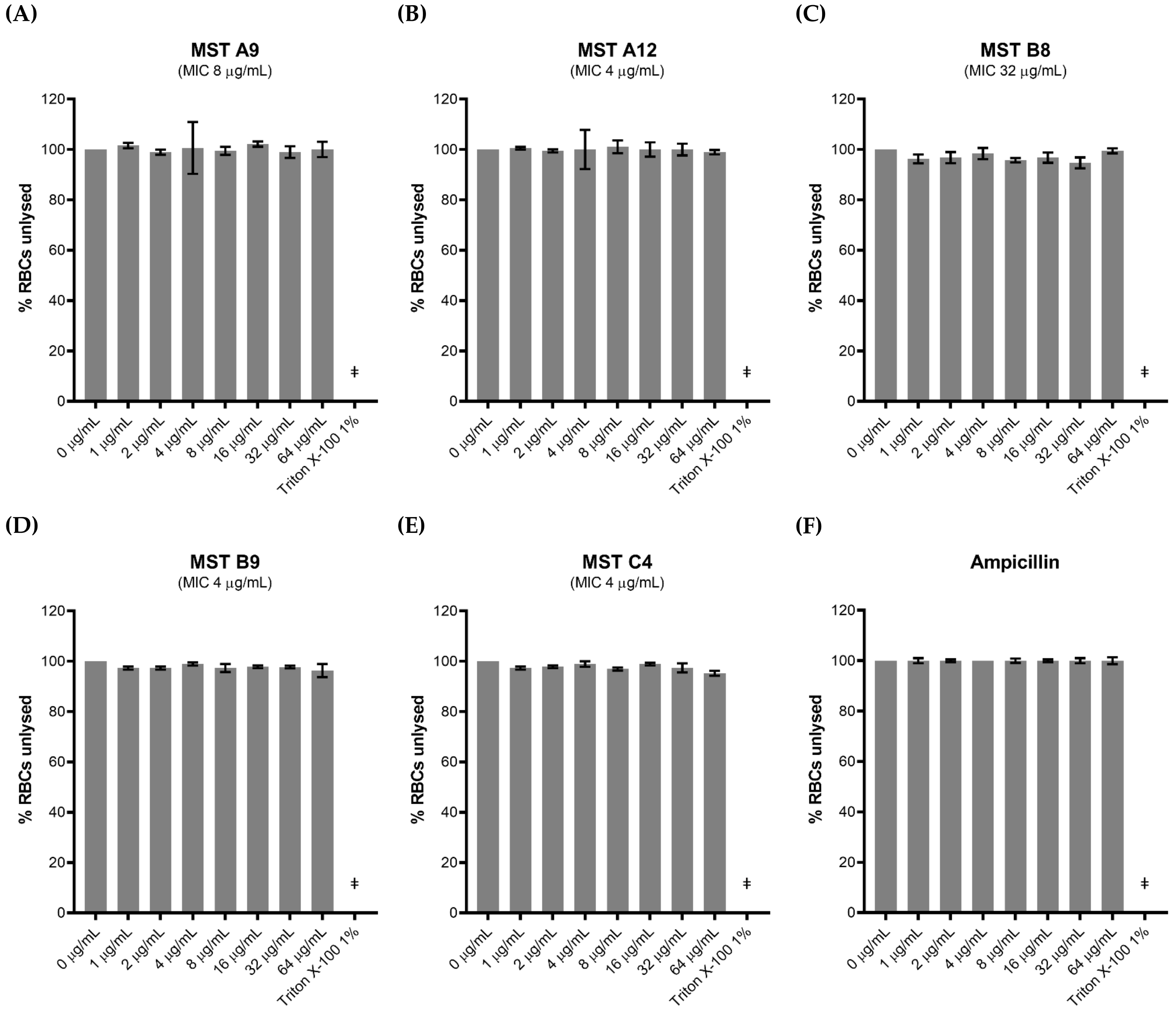

Figure S5. The MST compounds (A-E) and ampicillin (F) display no haemolytic activity.

Freshly washed human RBCs in PBS solution (137 mM NaCl, 2.7 mM KCl, 1.46 mM KH_2PO_4, 8.1 mM NaH_2PO_4, pH 7.4) was exposed to 2 µL MST compounds with concentrations ranging from 0 to 64 µg/mL in 1% (v/v) DMSO. A 1% (v/v) Triton X-100 solution was used to indicate complete RBC lysis (‡). Ampicillin (0 - 64 µg/mL) was used as example of drug that does not cause RBC lysis. The assays were performed in quadruplicates. The plates were incubated at 37 °C while constantly shaking at 100 rpm for 1 h. Intact RBCs were removed by centrifugation and the presence of haemolytic products in the supernatant were determined by measuring the absorbance at A_{450} nm. The results are presented in mean ± SEM. Statistical analysis was performed using one-way ANOVA and indicate no statistically significant change in RBC lysis ($p > 0.05$).

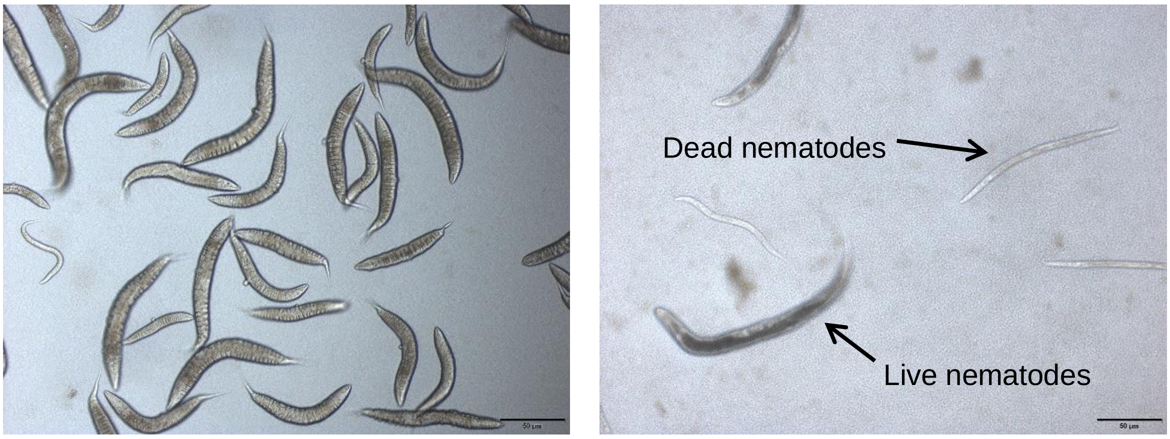

Figure S6. *Caenorhabditis elegans* nematodes (no treatment) viewed under the light microscope at time 0 h (left) and 72 h (right).
A standardized density of nematodes were harvested and cultured in optimized growth media. At every 24 h for 72 h, live vs dead nematodes were counted under the light microscope at 400× magnification. Observations for live vs dead nematodes can be clearly differentiated with its morphology as seen in a representation of the microscopy image shown above. Dead nematodes are thin and long with 'needle-like' appearance. The MST compounds were tested at 2×, 4× and 8× their MIC values to study its cytotoxicity on an *in vivo* model. The scale bar is 50 μm.

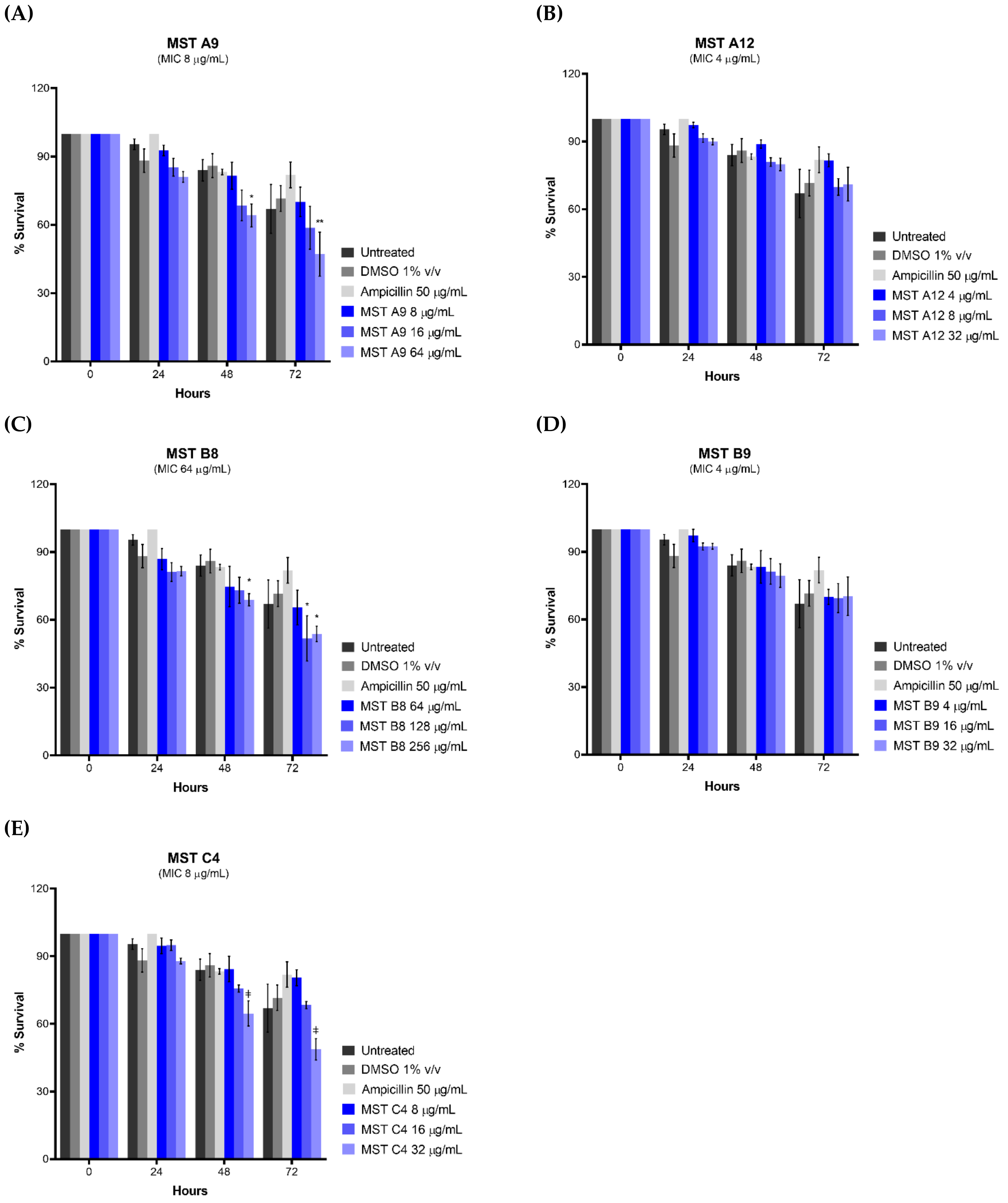

Figure S7. The MST compounds (A-E) did not display cytotoxicity in *C. elegans* nematodes up at 2× their MIC values.

C. elegans nematodes were cultured on nematode growth media, with *E. coli* as its primary source of nutrient. Newly harvested nematodes were investigated for toxicity in the presence of the MST compounds at 2×, 4× and 8× their MIC values for a timespan of up to 72 h. The nematodes were counted under a light microscope at 400× magnification and the live nematodes at 72 h was indicated as a fraction of the starting number of nematodes (percentage survival). The

results are presented as the mean ± SEM. Statistical analysis was performed using two-way ANOVA. The asterisks (*) represent statistical significance $p < 0.05$ and the alveolar (ǂ) represents statistical significance $p < 0.005$.

© 2020 by the authors. Submitted for possible open access publication under the terms and conditions of the Creative Commons Attribution (CC BY) license (http://creativecommons.org/licenses/by/4.0/).
